# Evolution of the reptile spine reveals independent trajectories to axial skeletal complexity in amniotes

**DOI:** 10.1038/s41467-026-72071-x

**Published:** 2026-04-16

**Authors:** Lucy E. Roberts, Jason J. Head

**Affiliations:** 1 Department of Zoology and University Museum of Zoology, University of Cambridge, Cambridge, UK; 2 Natural History Museum, London, UK

**Keywords:** Palaeontology, Herpetology, Phylogenetics

## Abstract

The evolution of complex, highly regionalized and heterogeneous axial skeletons within amniotes has traditionally been considered a unique characteristic of Pan-Mammalia, with limited complexity evolving independently in some reptiles. The ability to resolve axial skeletal evolution across Amniota remains restricted due to the lack of studies comprehensively exploring axial skeletal complexity through deep time outside of Pan-Mammalia. Here, we combine 3D geometric morphometrics of vertebral morphology with maximum likelihood model testing in a phylogenetic context to quantify regionalization and morphological heterogeneity in the presacral vertebral column of reptiles and representative tetrapod outgroups. We recover evidence for the evolution of four regions at least four times independently within amniotes, highly heterogeneous axial skeletal anatomies in archosaurs, and no evidence for uniquely complex vertebral anatomies in mammals. Heterogeneity is positively associated with body size in most reptile clades except for theropod dinosaurs, which also reduce regionalization toward the avian crown. The evolution of volancy is correlated with high heterogeneity, potentially associated with functional modularity of the cervical and dorsal regions. Our results indicate that complex axial skeletons arose independently and repeatedly in reptiles in addition to mammals, variably associated with the remarkable diversity in size, body form, function, and ecology across amniotes.

## Abstract (web summary)

Complex axial skeletal morphology has evolved in mammals and some reptiles. Here the authors combine morphometrics with a maximum likelihood model to find that axial heterogeneity is associated with body size in most reptiles and with flight more generally, pointing to multiple independent origins of axial complexity.

## Introduction

The axial skeleton, encompassing the vertebral column and dorsal ribs, is divided into discrete regions identified by morphological characteristics^1,2^. These regions are associated with functions including locomotion and respiration^3–13^, and are patterned embryonically by the expression of *Hox* genes in the precursors of vertebrae and ribs before and during somitogenesis^14–16^. Evolutionary trends in axial skeletal complexity, defined as the total number of regions (regionalization^2,3^) and the total amount of variation between all vertebral elements (heterogeneity^4^), have historically been interpreted as increases from a homogenous, simplified system in fishes and stem tetrapods to a highly regionalized, complex axial skeleton in mammals within Amniota^4,17–19^. However, recent studies have demonstrated higher than expected degrees of regionalization in some reptiles and fishes^3,20,21^, resulting in competing hypotheses in which regionalized axial skeletons are either ancestral to Amniota^3,21^ or arose within the clade, with a maximum of five presacral regions restricted to crown mammals^4^. Specifically: Overlap between *Hox* expression boundaries and morphometric regions in the skate *Leucoraja* indicates developmentally-regulated regionalization similar to tetrapods in at least one chondrichthyan^21^; Quantified morphology indicates that snakes retain axial regionalization contrary to hypotheses of developmental deregionalization in elongate body-form evolution, and that *Hox*-mediated regionalization is an inherited character of amniotes^3^, and; Vertebral complexity reconstructed across Pan-Mammalia reveals low regionalization and heterogeneity along the mammal stem with independent increases in the reptile and mammal crowns^4^. The hypotheses of these studies are based on limited taxon sampling. Head and Polly^3^ focused primarily on squamates, whereas Jones et al. ^4^ restricted sampling of Reptilia to ten extant lepidosaurs and three extant crocodylians^4^. Only Criswell et al. employed 3D geometric morphometric methods to precisely quantify vertebral shape in three dimensions^21^. As a result, the evolution of axial skeletal complexity in Reptilia, and its implications for understanding the origins of complexity in tetrapods or amniotes remains poorly known.

**Fig. 1:**
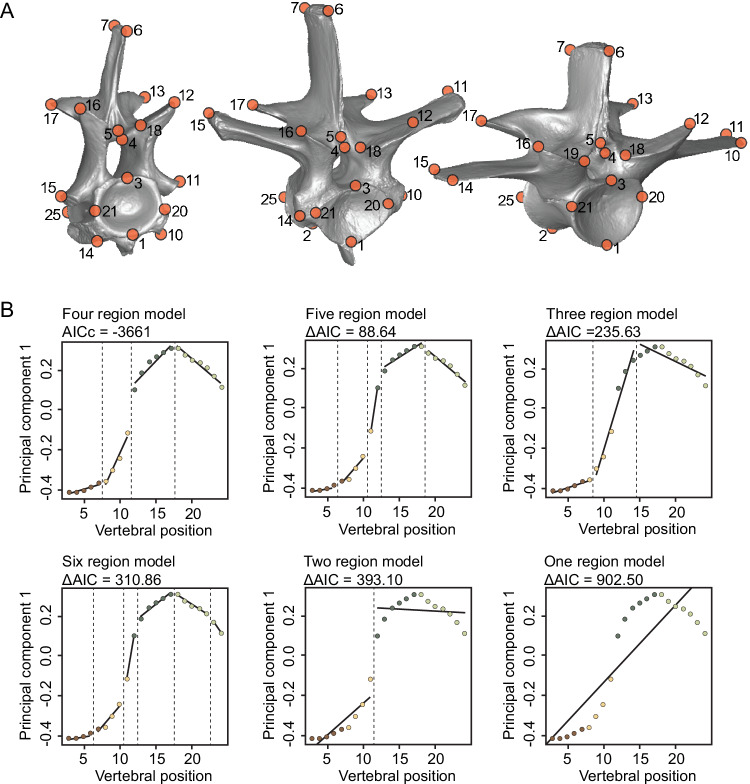
Morphological variation in the presacral vertebral column of *Alligator mississippiensis.* **A** Cervical (vertebra 5), thoracic (vertebra 11), and lumbar (vertebra 22) vertebral elements in right anterolateral view. Numbered landmarks were used to quantify vertebral shape. **B** The first principal component of shape change in the presacral vertebral column against vertebral position with the best-fit segmented linear regression models for each hypothesised number of regions from one to six. Source data are provided as a Source Data file.

**Fig. 2:**
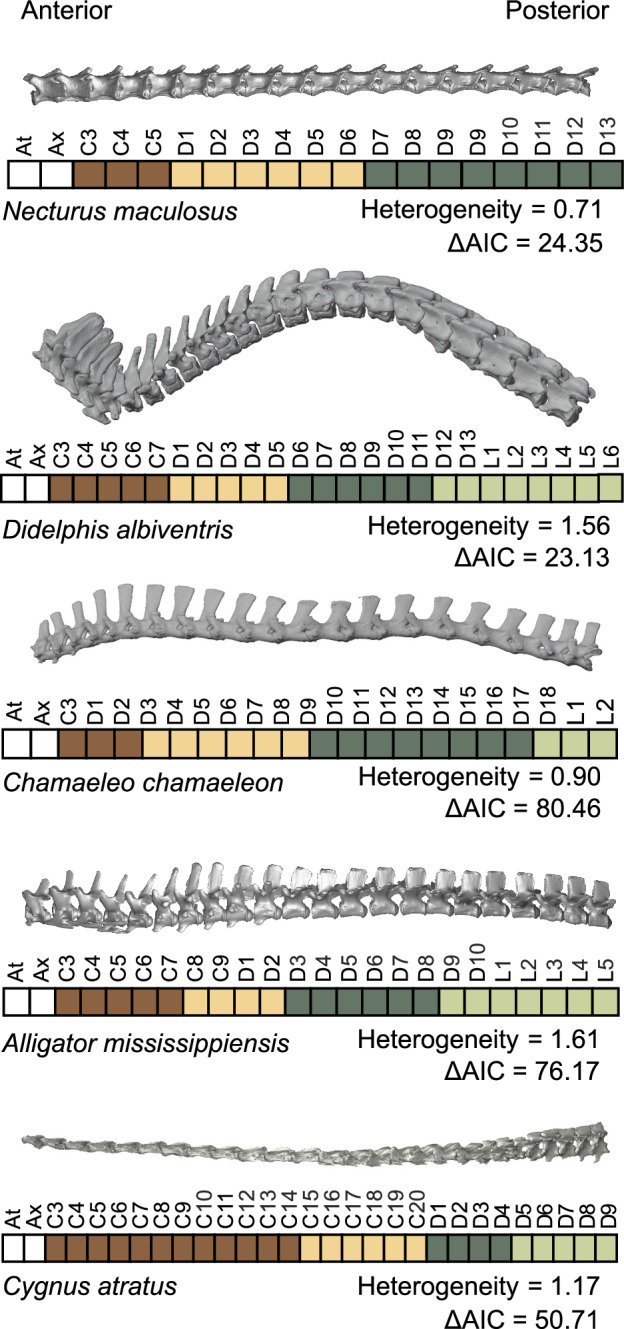
Presacral vertebral columns and modelled number of regions for representative taxa of major tetrapod clades. Boxes represent vertebrae, colours represent regions within the presacral axial skeleton for the best-fit model of each taxon. Heterogeneity values and AIC are for best-fit models. Abbreviations: At, atlas; Ax, axis; C, cervical; D, dorsal, L, lumbar. Source data are provided as a Source Data file.

To comprehensively reconstruct the evolution of axial skeletal complexity across Reptilia, we quantified vertebral shape for representative taxa of all reptile clades, including stem and crown taxa. We modelled intracolumnar heterogeneity as the mean of the Procrustes distances calculated for each pair of elements in the column, and modelled regions as morphological gradients in the presacral vertebral column using segmented linear regression on shape variables derived using 3D geometric morphometrics (Fig. 1, Supplementary Table 1), and maximum likelihood model testing to select the best model for each specimen (Fig. 2). We classified model fit using the corrected Akaike Information Criterion (AICc) to penalize against overparameterization, which allows models with different segment and hypothesised region counts to be directly compared. We carried out these analyses in R (ver. 4.3.2)^22^ using the regions package, developed by Jones et al. ^4,23^, based on Head and Polly^3^. We use *regions* because it enables extension of the methodology for 3D geometric morphometric data rather than 2D geometric morphometric data^3^ or linear measurements^4,23,24^. We excluded the atlas-axis complex from our analyses, because it is a ubiquitous functional and morphological unit among tetrapods. We analysed the presacral vertebral columns of 96 taxa across Pan-Reptilia and representative outgroups to test hypotheses and assumptions regarding axial skeleton evolution across Amniota (Supplementary Fig. 1). We sampled the majority of extant reptile clades, including crocodilians, birds and lepidosaurs, but excluded turtles and snakes. Dorsal axial skeletal morphology of turtles cannot be quantified using the homologous landmarking scheme of our study as it is incorporated within the rigid carapace. Axial skeletal regionalization in snakes has been comprehensively examined within prior works^3,4,7^ and sampling whole vertebral columns with greater than 40 vertebral elements gives statistically unstable results (see methods). The evolution of axial skeletal complexity in mammals has been analysed^4,7,23^ and provides a robust comparative framework for modelling patterns in reptiles. We include a representative sample of higher-order mammal clades to model ancestral states for amniotes and to compare results of our methods with previous studies^4,7,23^. We examined extant representatives of Rhynchocephalia, Squamata, Crocodylia and Aves, as well as stem reptiles, stem archosaurs, and stem Aves (Supplementary Fig. 1). To examine the relationship between axial skeletal complexity and ecology, we sampled taxa representing a wide diversity of locomotor ecologies in Reptilia, from fully aquatic to volant. Our analytical criterion for including fossils requires mostly complete axial skeletons of individuals that can be examined in all views and orientations. As a result, we were forced to exclude several key fossil taxa such as captorhinomorphs, pareiasaurs and procolophonids due to preservational limitations and specimen availability (see discussion in Supplementary Methods).

We conducted sensitivity analyses to test the repeatability of landmarking and the regions analysis, and to test the effects of intraspecific variation, the number and placement of landmarks, the number of principal components used in the regions analysis, the effect of incomplete specimens or damage to individual elements, and variation in phylogenetic hypotheses used (Supplementary Methods, Supplementary Figs. 2–7, 9 and Supplementary Tables 2–4, 9). In order for specimens, fossil or otherwise, to be suitable for the methods herein the presacral series must include most elements, with a tolerance of 1–2 missing vertebrae which cannot include the first or last element, and be preserved in three dimensions. To test for potential differences in model selection for taxa with different segment counts, where additional vertebral elements constitute additional model parameters, we ran supplementary standardized models by sampling every 5% of the column as a sensitivity analysis^3^ (Supplementary Fig. 8). We placed results in a phylogenetic context using a composite amniote tree topology^25–42^ (Supplementary Fig. 1 and Supplementary Table 5) and conducted evolutionary model testing and phylogenetic comparative methods including continuous ancestral state estimation and stochastic character mapping. We frame our results in the wider context of body size and ecology to assess the potential drivers and constraints on axial skeleton diversity across Pan-Reptilia. We use ecological categories specific to locomotory mode (e.g. volant, arboreal) as this aspect of ecology is functionally associated with vertebral heterogeneity in multiple clades^5,43–45^.

## Results

**Fig. 3:**
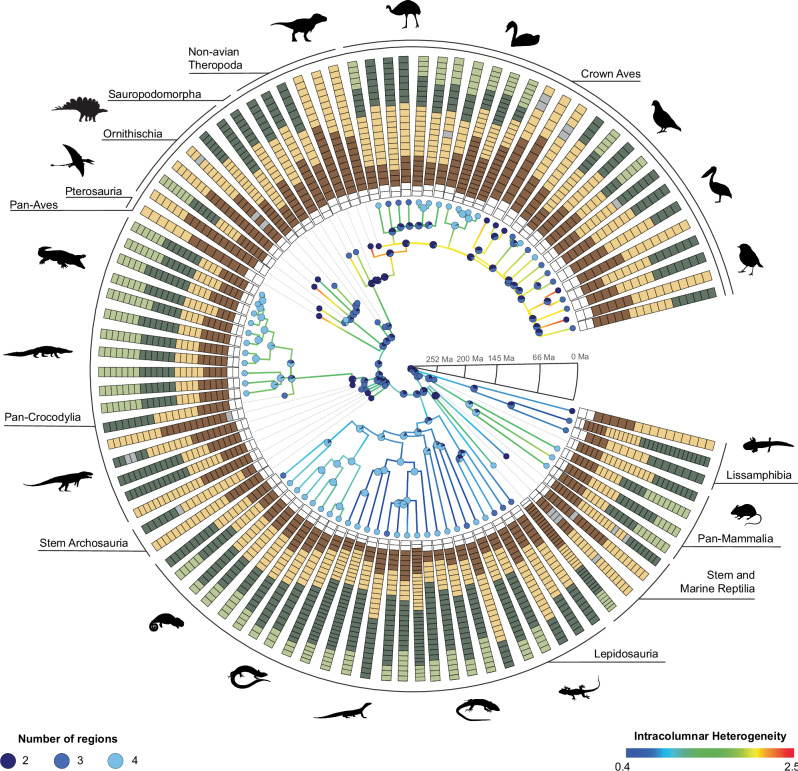
Modelled vertebral column regionalization and heterogeneity across sampled tetrapods. Branch colours represent intracolumnar heterogeneity for sampled taxa and ancestral states. Coloured dots at branch tips represent the best-ft modelled number of regions for sampled taxa, pie charts at nodes represent the proportional likelihoods of region numbers at that node. Boxes represent vertebrae, colours represent regions within the presacral axial skeleton for the best-fit model of each taxon. Silhouettes were accessed via PhyloPic and have all been dedicated to the public domain under a CC0 1.0 Universal Public Domain Dedication Licence.

**Fig. 4:**
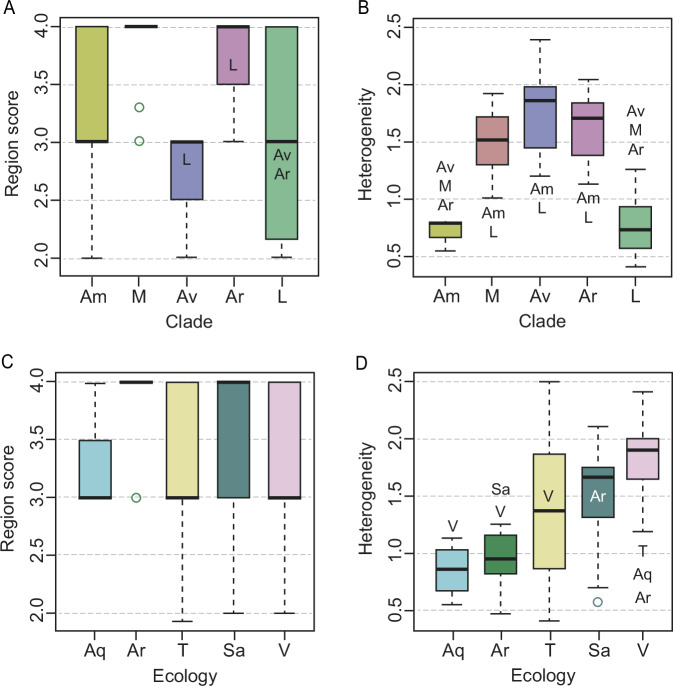
Region score and intracolumnar heterogeneity categorised by clade and ecology. Abbreviations written on boxes represent a significant difference in the mean between the categories represented by the box and by the abbreviation. **A** Distribution of region scores by clade. **B** Distribution of heterogeneity by clade. **C** Distribution of region scores by ecological category. **D** Distribution of intracolumnar heterogeneity by ecological category. Clades: Am = Lissamphibia (*n* = 3), M = Pan-Mammalia (*n* = 4), Av = Crown Aves (n = 29), Ar = non-avian archosaurs (Pan-Crocodylia + Stem Aves, *n* = 34), L = Lepidosauria (*n* = 26). Ecological categories: Aq = fully aquatic (*n* = 4), Ar = arboreal (*n* = 10), T = terrestrial (*n* = 44), Sa = semi-aquatic (*n* = 21) and V = volant (*n* = 17). Boxplots show the median (central line), interquartile range (box; 25th–75th percentiles), and whiskers extending to the most extreme values within 1.5 × the interquartile range. Points beyond the whiskers represent outliers. Differences between groups were assessed using one-way ANOVA (two-sided), followed by Tukey’s honest significant difference (HSD) test for multiple comparisons with family-wise error adjustment. For region scores by clade, significant pairwise differences were observed between Lepidosauria and Crown Aves (*P* = 0.013397) and between Pan-Archosauria (excluding Crown Aves) and Lepidosauria (*P* = 0.00149). For intracolumnar heterogeneity by clade, significant differences were detected between Lepidosauria and Crown Aves (*P* < 0.0001), Lissamphibia and Crown Aves (*P* = 0.000030), Pan-Mammalia and Lepidosauria (*P* = 0.001358), Pan-Archosauria (excluding Crown Aves) and Lepidosauria (P < 0.0001), Pan-Mammalia and Lissamphibia (*P* = 0.03025), and Pan-Archosauria (excluding Crown Aves) and Lissamphibia (*P* = 0.000228). All other comparisons were not statistically significant (adjusted *P* ≥ 0.05).

**Fig. 5:**
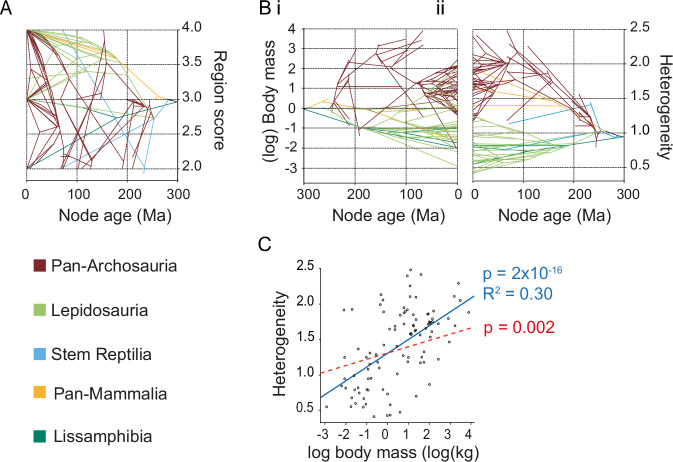
Evolution of axial skeletal complexity and body mass. **A** Phenogram showing tip and modelled region scores across tetrapod phylogeny over time, **B** Phenograms showing tip and modelled node states across tetrapod phylogeny over time for **i** body mass, **ii** Intracolumnar heterogeneity. **C** Regressions of body mass against intracolumnar heterogeneity across Pan-Reptilia. Blue solid line represents ordinary least-squares regression, red dashed line represents phylogenetic least-squares regression under an OU model. The association between heterogeneity and log-transformed body mass was evaluated using ordinary least-squares regression (lm) and phylogenetic generalized least-squares (PGLS) regression. Tests were two-sided. Exact *P* values are reported in the figure. No correction for multiple comparisons was applied.

We find evidence of at least four independent trajectories to a four region presacral axial skeleton across Amniota. We model a 58% probability of a two-region ancestral state in the hypothetical ancestor uniting our whole sample of tetrapods, and a 70% probability of a three-region ancestral state in the hypothetical common ancestor uniting amniotes^4^ (Fig. 3 and Supplementary Table 6). At the node uniting Pan-Reptilia we recover a 90% probability of a three-region ancestral state (Supplementary Table 6). We model high probabilities of three-region states ancestrally for Pan-Mammalia, Lepidosauria, Pan-Crocodylia and Pan-Aves, and independent increases to four regions across each major reptile clade (Fig. 3 and Supplementary Table 6). Four regions are conserved amongst all extant crocodylians, the majority of extant lepidosaurs, and approximately one-third of extant birds. Four modelled regions in extant lepidosaurs and crocodylians is consistent with previous studies^3,4^, but we find no evidence for five regions in mammals^4^. We also model a decrease in regionalization ancestrally to Crown Aves and recover high levels of variability from two to four regions among extant birds. We recover significantly higher mean regionalization in lepidosaurs compared to birds, and significantly lower regionalization in lepidosaurs relative to non-avian archosaurs (Fig. 4A and Supplementary Table 8). Heterogeneity is also variable, but there are distinct differences in this variability across phylogeny compared to regionalization (Figs. 3 and 5A, Bii). Intracolumnar heterogeneity is significantly higher in mammals and archosaurs than in lepidosaurs or sampled amphibians^4^ (Fig. 4B and Supplementary Table 8).

We fit evolutionary models to region and heterogeneity scores to examine the evolutionary tempo and mode of axial skeletal complexity and to test whether these metrics evolved independently through time. We tested nine competing models: Passive, Brownian Motion (BM, random walk), BM with active trend, evolution towards a single evolutionary optimum (Ornstein-Uhlenbeck, OU), and six different multiple-optima OU models to test for shifting adaptive optima, which indicate stabilizing selection toward an optimum, or a change in the adaptive landscape across a lineage. Our results reject a Brownian (random walk) model and instead support stepwise shifts in the evolution of both regionalization and heterogeneity (Supplementary Table 7). Evolutionary trait modelling across Pan-Reptilia indicates that heterogeneity and regionalization evolved under a multiple optimum OU model, consistent with stepwise shifts in complexity modelled previously in the mammalian vertebral column^46^. We modelled a shift toward increased heterogeneity within Pan-Archosauria prior to the divergences of Crown Archosauria (Fig. 5Bii and Supplementary Table 7). Lepidosaurs either retain or slightly decrease ancestral levels of intracolumnar heterogeneity. We modelled a shift towards increased regionalization within Lepidosauria (Fig. 5A and Supplementary Table 7). Modelled optimum regionalization increases from three prior to the divergence of lepidosaurs and archosaurs to four across Lepidosauria. Regionalization across Pan-Archosauria varies across the entire range of modelled values, from two to four.

We found no significant relationship between regionalization and either ecological locomotory mode (Fig. 4C) or body size (Supplementary Fig. 12), but intracolumnar heterogeneity is associated variably with locomotor ecology (Fig. 4D) and body mass (Fig. 5C). Linear regression indicates a significant, positive relationship between intracolumnar heterogeneity and body mass across Reptilia (*p* = 1.5 × 10^−8^) (Fig. 5C). This significant relationship is maintained when modelled using phylogenetic generalised least squares (PGLS) under the best-fit OU model of evolution (*p* = 0.002) (Fig. 5C and Supplementary Table 7). Deviations from the overarching pattern of linear increases in heterogeneity with size have the potential to reveal additional functional variation in heterogeneity. We quantified these deviations as residual values of heterogeneity after regression against (logged) body mass (Supplementary Fig. 10). We found negative residual heterogeneity in anguimorph squamates; mean heterogeneity is lower than expected based on the relationship between heterogeneity and body size for the lizards *Varanus* and *Heloderma*. We modelled positive residual heterogeneity across most volant birds; mean heterogeneity is higher than expected based on the relationship between heterogeneity and body size alone. Volant taxa have significantly more heterogeneous presacral vertebral columns compared with aquatic, arboreal and terrestrial taxa, and heterogeneity is significantly higher in semi-aquatic taxa compared with arboreal species (Fig. 4D and Supplementary Table 8).

## Discussion

The evolutionary history of the axial skeleton has historically been considered to include a trajectory toward uniquely high regionalization in crown mammals^4,17–19^. Our results enable us to reject this view. Not only do we find that reptiles are just as highly regionalized as mammals, but our results demonstrate that four-region anatomies evolved from three-region ancestors independently within Lepidosauria, Pan-Crocodylia, and Pan-Aves. We also found no evidence for five regions in our sample of mammals, in contrast to analyses that maximise region number by reducing the proportion of shape data used to model regions^4,23^ (see discussion in Supplementary Methods). We additionally found that regionalization and heterogeneity evolved independently, which is consistent with previous evolutionary modelling assessing regionalization and heterogeneity across Pan-Mammalia^4,46^. Taken together these findings indicate that the evolutionary independence of regionalization and heterogeneity is present across Amniota.

**Fig. 6:**
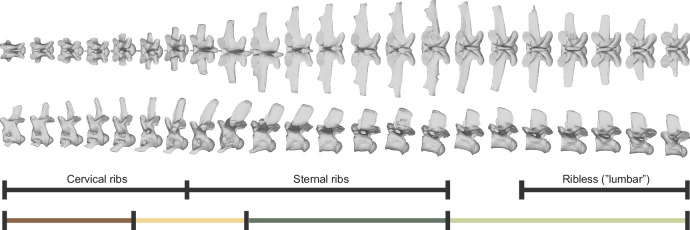
Comparison of rib-based and morphometric regions in *Alligator mississippiensis*. Vertebrae 3–24 of *Alligator mississippiensis* in dorsal and left lateral views, alongside traditional regional divisions (top) based on rib presence and articulation with the sternum, and distribution of morphometric regions (bottom).

Our results highlight the importance of precise quantification of morphology in analyses of phenotypic evolution. This analysis, and previous studies on regionalization in squamates and chondrichthyans demonstrate cryptic levels of regionalization that cannot be qualitatively discerned by eye^3,21,47^, and are likely to be missed by linear morphometrics^4^, where the important axes of variation have been chosen a-priori, are less able to accurately represent the shape of complex structures^48,49^, and can overinflate the allometric component of shape^50^. Sensitivity testing demonstrates that a reduction in the resolution of input landmark data, or a reduction in the number of principal components used to model regionalization, can increase or decrease the modelled number of regions in less heterogeneous taxa (Supplementary Methods, Supplementary Figs. 3, 4). Traditionally, region boundaries have been discerned by visible shifts in discrete vertebral characters or by the presence, absence and morphology of the ribs and limb girdles^51^; however, regionalization of vertebral elements and ribs is conferred differently during development; the vertebrae and proximal portion of the ribs are patterned by the colinear *Hox* code, but *Hox* gene function in the lateral plate-derived limb girdles, sternum and sternal ribs is independent of somite-derived patterning and is not colinear^52^. Conservation of regionalization in the primaxial portion of the ribs has been demonstrated in squamates^53^, but the regional identity of vertebrae cannot be discerned using the presence or absence of connection to the sternum. This is apparent when comparing regions modelled herein with traditional regional divisions in *Alligator mississippiensis* (Fig. 6). Regions modelled using 3D geometric morphometrics recover changes in the position of the rib attachment surfaces, which does not occur in direct concert with shifts in distal rib morphology (Fig. 6). For assessments of regionalization to be meaningful and applicable to understanding development, it is necessary to quantify the complex shape of vertebral elements as accurately as possible, without prior assumptions regarding the key axes of variation or position of region boundaries.

### Ecology and function mediate axial skeletal complexity

We do not recover a relationship between regionalization and phylogeny, body size or ecology in reptiles (Fig. 4A, C and Supplementary Fig. 12). Increased intracolumnar heterogeneity, however, is associated with larger body sizes across Pan-Reptilia (Fig. 5C). Generally, lepidosaurs are smaller and have more homogeneous vertebral columns, whereas archosaurs, with the exception of most birds, are larger, and have more heterogeneous axial skeletons (Fig. 5B, C). We propose that, at larger body sizes, biomechanical pressures on the vertebral column vary more strongly with vertebral position, increasing bending stresses and necessitating variation in morphological adaptation to these stresses in different portions of the column^54,55^. Functional and ecological constraints on vertebral morphology potentially overcome this biomechanical scaling and result in high heterogeneity even at lower body sizes, including high residual heterogeneity in birds (Supplementary Fig. 10). Pervasively amongst non-avian dinosaurs and palaeognath birds^56^, intracolumnar heterogeneity follows the generalized reptilian pattern—heterogeneity approximates values expected by body mass scaling alone. Volant taxa however, have elevated heterogeneities relative to their body size (Supplementary Fig. 10). Elevated residual heterogeneity amongst most sampled maniraptorans reflects morphological differentiation within and between the cervical and dorsal regions (Supplementary Fig. 9B). The structure of the dorsal axial skeleton provides a frame for the flight apparatus and facilitates efficient gas exchange^57,58^. This specialization, alongside functional specialisation of the forelimb for flight, limits the functional versatility of the dorsal skeleton^59^. Instead, morphological variation within the avian cervical skeleton is an adaptation for increased functional diversity of the neck^60–62^, contributing to higher heterogeneity throughout the presacral vertebral column in birds relative to other reptiles, and at low body sizes (Fig. 3 and Supplementary Fig. 10).

Heterogeneity, regionalization, and the position of region boundaries are almost invariant amongst extant crocodylians (Fig. 3). High body mass relative to many other reptiles potentially contributes to relatively high heterogeneity across the clade, however, discrete characteristics that delineate modelled regions are present, including a distinct cervical vertebral morphology and a ribless lumbar region (Fig. 6 and Supplementary Fig. 9C). In every sampled extant crocodylian we recover a region boundary between vertebrae 11 and 12 (Fig. 3). Across this boundary the angle between the diapophysis and parapophysis changes markedly^51^ (Fig. 6). Empirical evidence demonstrates that in crocodylians during costal ventilation the angle between the parapophysis and diapophysis influences the motion of the ribs^63^. Previously this has been reported as a difference between the anterior thoracic vertebrae and the remaining elements of the thoracic region^63^. Regions modelled here however demonstrate that these two vertebrae occupy a separate region alongside the posterior two vertebrae traditionally designated as cervical (Fig. 6). This anatomical separation unites the elements in each of these two regions morphologically and functionally. The consistency of this boundary across all sampled crocodylians, alongside their functional importance, suggests that the position of this boundary is constrained amongst crown crocodylians by respiratory function.

Across Lepidosauria, heterogeneity decreases from degrees ancestral to Pan-Reptilia, and remains low despite highly regionalized anatomies, and regardless of body size (Figs. 3, 4 and 5B). Low heterogeneity despite high regionalization is consistent with previous findings regarding conservation of *Hox*-mediated regionalization throughout Squamata, via cryptic regionalization i.e. low heterogeneity between regions^3^. Low heterogeneity potentially reflects a lack of functional specialisation in vertebral shape relative to birds and crocodiles. Within Squamata we model elevated heterogeneity and residual heterogeneity amongst iguanians (Supplementary Fig. 10). This is observable when comparing specimens of iguanian taxa with non-iguanian squamates (e.g. *Chamaeleo chamaeleon* and *Nephrurus asper*, Supplementary Fig. 9A). Furthermore, Iguania is the only squamate clade across which elongate, limbless body forms have not evolved^10^, potentially reflecting a reduction in developmental lability relative to other squamates. Regional heterogeneity in chameleons has been linked to arboreality^5^; relatively higher heterogeneity in iguanians may therefore be associated with functional and ecological adaptation.

### Summary

Uniquely high axial skeletal complexity has previously been held as a defining characteristic of mammalian anatomy and evolution^4,17–19,24^. We find no evidence for higher heterogeneity or regionalization in mammals relative to reptiles. Instead, our results demonstrate that most extant reptiles are just as regionalized as extant mammals, and we recover high axial skeletal heterogeneity among archosaurs within Reptilia. We find that presacral vertebral columns made up of four regions have arisen at least four times independently across Crown Amniota, and that intracolumnar heterogeneity is associated pervasively with body mass across Pan-Reptilia but functional and ecological influences on axial skeletal morphology can result in high heterogeneity even among smaller body sizes. Evolutionary histories of complexity across Pan-Reptilia, and potentially Amniota, likely reflect the variable presence and absence of functional and ecological influences rather than a single monotonic trend of increasing complexity associated with the evolution of mammalian physiology. Variation in axial skeletal complexity across Pan-Reptilia has contributed to the radiation of a remarkable diversity of function, form, and ecology both in the present and through deep time.

## Methods

### Taxon sampling and phylogenetic hypothesis

We attained all necessary permissions for scan and specimen access. See Supplementary Data 1 for details on specimen and scan providence. We sampled representatives of the majority of living reptile clades. This includes specimens that we directly digitised from multiple museum collections and for which scan data was available remotely (Supplementary Data 1). We could not include turtles because the incorporation of their dorsal axial skeleton into the rigid carapace of the shell limits the ability to segment the dorsal vertebrae and to recognize unambiguously homologous morphometric landmarks. We sampled extant and fossil representatives of the primary divisions within Crown Reptilia: Lepidosauria and Archosauria. Within these clades we examined extant representatives of Rhynchocephalia, Squamata, Crocodylia and Aves. We sampled fossil stem taxa for Reptilia, Archosauria, Crocodylia, and Aves (Supplementary Fig. 1). Due to the required level of specimen completeness, no fossil lepidosaur specimens were available for study. Taxon selection was designed to sample the ranges of body size and locomotory mode for each clade; however proportional taxonomic richness was not possible for the two most speciose clades (Crown Aves, Squamata). For example, in Aves we include both the largest terrestrial, non-volant species and some of the smallest volant taxa. Axial skeletal regionalization in snakes and snake-like squamates has been previously demonstrated^3^, and we did not include them in this study. Our small sample of mammals is designed to replicate larger-scale trends seen in previous analyses^4,7,8^ and provide comparative data for evolutionary modelling.

The phylogenetic hypothesis we used is a consensus tree derived from multiple hypotheses. We used a phylogenetic framework of Pan-Reptilia and added additional taxa which were not included in the original topology (Supplementary Table 5). We placed *Dolichorhynchops osborni* based on the interrelationships of Sauropterygia relative to Lepidosauria and Archosauria according to Neenan, Klein and Scheyer^25^. Due to competing hypotheses regarding the taxonomic affinity of sauropterygians and choistodires we conducted sensitivity analyses to test the impact of alternative phylogenetic placements^64–70^ (Supplementary Fig. 11 and Supplementary Table 9). We placed *Machaeroprosopus pristinus* based on the position of Phytosauria according to Nesbitt et al. ^28^. We resolved relationships across Squamata according to Zheng and Wiens^26^, and across Aves according to Prum et al. ^40^. These topologies were derived using molecular data, rather than morphological data. We chose the molecular topologies here as these data are less likely to be affected by evolutionary convergence; topologies based on molecular data are generally considered more reliable estimates^71–74^. To temporally calibrate the compiled phylogeny, we used the bin_cal3TimePaleoPhy function in paleotree (ver. 3.3.25)^75^ in R to generate 100 different temporally calibrated phylogenies (paleotrees) for estimating ancestral states. This function uses first and last appearance fossil data and estimates of branching, extinction, and sampling rate. We downloaded first and last appearance data from the Paleobiology Database, using the genus and species names of fossil taxa in the compiled phylogeny. Branching, sampling, and extinction rates were estimated using the make_durationFreqDisc function, also in paleotree. We downloaded minimum node time calibrations for extant taxa from timetree.org^41,42^. We produced a consensus phylogeny from the 100 produced paleotrees using the consensus.edges function in phytools^76^, which calculates the mean edge length for each branch across all 100 input trees.

### Data collection

We digitized vertebral morphology based on micro-CT and surface scans. Micro-CT scanning of specimens from the University Museum of Zoology, University of Cambridge, was conducted using a Nikon Metrology XT H 225 ST High Resolution CT Scanner using a 225 kV X-ray tube at the Cambridge Biotomography Centre, University of Cambridge. Volume construction from raw X-ray data was performed using CT PRO software (Metrix X-Tek, UK). We imported micro-CT data into AVIZO and used the segmentation editor to digitize vertebral elements as separate models. We digitized large ( > approx. 20 mm vertebral diameter) specimens using an Artec Space Spider blue-light scanner and processed 3D scan data using Artec Studio 13 Professional. Surface scanned specimens are housed in various museum collections in the UK and USA (Supplementary Data 1). We acquired additional micro-CT and surface scan data from multiple sources, including MorphoSource.org and OSF.io. Source data is available in Supplementary Data 1, details of data acquisition are available for each specimen on the appropriate site. Each digitized set of vertebral elements includes all presacral vertebrae exclusive of the atlas-axis complex, which is a consolidated morphological and functional unit across all amniotes. Similarly, the sacral vertebrae constitute a distinct unit. Some avian species have a fused portion of vertebrae, a notarium, immediately anterior to the synsacrum. Two taxa originally included in this analysis have notaria that are completely fused: *Gallus gallus* and *Meleagris gallopavo*. When these taxa there is no separation between the final two to five vertebrae. We produced models of vertebral elements for *G. gallus* using CT scans; the CT scans reveal no internal separation of the fused vertebrae. For this reason, the vertebrae are inseparable. The position of any division made in order to separate the vertebral elements would not be based on any morphological markers, and the resulting models would lack any homologous structures on the anterior or posterior faces of the other pre-synsacral vertebrae, as well as lacking any zygapophyses. We are unable to utilise these fused vertebral elements in the geometric morphometric analyses included here. As this portion of the pre-synsacral vertebral column represents a distinct series that cannot be incorporated into the regionalization analysis, the taxa that have a fused section are excluded from further analysis. In addition to anatomical data we collected body mass data from the literature (Supplementary Data 2). For fossil taxa we used published estimates of body mass, compiling masses from as few sources as possible to limit methodological variation. Body mass estimates were not available for all taxa sampled in the regionalization analysis: *Stereosternum tumidum, Champsosaurus laramiensis, Dolichorhynchops osborni, Mahajangasuchus insignis*, and *Eusaurosphargis dalsassoi* were not included in the body mass analyses. Before further analysis we log transformed all body mass values.

### Regionalization analysis

We modelled regionalization using the regions^4^ package in R by applying segmented linear regression to shape variables that describe intracolumnar morphological variation, derived from 3D geometric morphometric analysis of vertebral anatomy^3,4^. We used 25 3D landmarks to describe vertebral shape in dorsal-ventral, anterior-posterior, or medial-lateral directions (Fig. 1A and Supplementary Table 1). We carried out extensive sensitivity testing to determine the number and placement of these landmarks (Supplementary Methods, Supplementary Figs. 2–4). We designed our methodology with no prior assumptions of anatomical transitions or traditional, expected regionalization such that out analyses are unbiased. All the landmarks used here are Type II. Landmarks on the midline of vertebrae encompass within-column variation in the height and depth of the centrum and neural arch. Landmarks on the lateral margins of vertebrae encompass variability in the morphology of zygapophyses. Landmarks on rib attachments capture their variable position relative to each other and the rest of the vertebral element. We measured shape covariation using Procrustes superimposition, which calculates the residual variation between homologous landmark coordinate points after landmark constellations are rotated, translated, and scaled to a common centroid size^77,78^. A unidimensional measure of covariation, the Procrustes Distance is calculated as the square root of the sum of squares of coordinate distances between constellations. Additionally, Principal Components Analysis of the resulting covariance matrix provides multidimensional shape variables for each specimen which can be analysed using ordination and statistical techniques^79–82^. We quantified intracolumnar differences in vertebral shape along the presacral vertebral column of each taxon. We uploaded 3D models of vertebrae into IDAV Landmark© software and applied landmarks to homologous positions. We exported all landmark coordinates and compiled them into a single TPS file which we then imported into the R environment. We used the procSym function in the Morpho package^83^ to perform the Procrustes superimposition. We then conducted a principal components analysis on the rescaled Procrustes landmarks using the plotTangentSpace function in geomorph (ver. 3.0.6)^84,85^ (Fig. 2). We conducted regionalization analyses using the *regions* package^4^. There is a more recent version of this code in the *morphoregions*^24,86,87^ package, however this is specifically built for use with linear morphometric data and is not statistically appropriate for use with geometric morphometric data. We plotted scores for each principal component in anterior-posterior order in the vertebral column in the regions package and applied a series of segmented linear regression analyses (SLR), where the number of regression lines is equal to the number of hypothesised regions (Fig. 1B). Segmented linear regression divides the domain into sections and fits individual regressions to each sub-domain^3,88^. The compileregions function in regions allows the SLR algorithm to fit a predetermined number of regressions and compares the fit of different regionalization models, represented by the corresponding number of regression lines, to the morphometric data. We included all principal components, which equals the number of vertebral elements analysed minus one, representing 100% of total intracolumnar shape variance. We carried out sensitivity analyses to test the effect of reducing principal component data, finding that for less heterogeneous specimens reducing the number of principal components used to model regions can produce unpredictable effects (see Supplementary Methods, Supplementary Fig. 4). Furthermore, we tested the PCOmax utility in regions which determines the number of principal components to use that will maximise the resulting number of modelled regions (Supplementary Fig. 4). We find no practical or theoretical benefit to arbitrarily reducing the number of principal components used in this analysis, unless one is aiming to inflate the modelled number of regions by ignoring more subtle intracolumnar gradients of shape (See Supplementary Methods). Cryptic or subtle regionalization has been identified in multiple groups^3,21^.

We set a maximum of six linear segments for analysis—one dimension greater than the maximum number of regions observed in any known amniote—to compare models from one to six regions. We then compiled the maximum-likelihood regression models for each potential number of regions using the modelselect function in regions. The natural logarithm is a monotonically increasing function, so log-transforming likelihood ensures that the maximum value given occurs at the same point as the probability function. The log likelihood is given by: *D* = 2ln(*λ*) Where *λ* is the likelihood ratio, which is the likelihood of a given regionalization model relative to the likelihood of the model given the principal component scores. From the six highest likelihood models from one to six regions we chose the best fit model using the corrected Akaike information criterion (AICc). The AICc scores relative model strength by assessing how well the model fits the given data, with a penalty weighted by parameterization^87^. Specifically, it is calculated for a given model by:

1 $${{\mathrm{AICc}}}=n\log ({{{\rm{\sigma }}}}2)+\tfrac{2K+2K(K+1)}{n \, - \, K\, -\, 1}$$

Where *n* is the number of variables, σ2 is the total residual sum of squares divided by *n*, and *K* is the number of estimated parameters, given by:

2 $$K=2\,{{r}}\, {{v}}+({{r}} {\mbox{-}}1)$$

Where *r* is the number of regions and *v* is the number of principal components. Using the AICc to choose the best-fit regionalization model appropriately penalizes against the phenomenon whereby models using greater numbers of regression lines will always fit the data better using likelihood alone. This best-fit model is the resulting hypothesis for the number of regions, giving the hypothesised position of region breaks for that taxon. We examined the difference in AICc values (ΔAICc) between the best and second-best model as an additional indicator of the relative strength of the best-fit model. A higher ΔAICc between models indicates greater strength of evidence for the best-fit model. There is no definitive minimum ΔAICc value to confirm a sufficiently strong model, but a ΔAICc between two to seven is high enough to consider rejection of the next-best fit model^87,89,90^. The relative strength of a model increases exponentially with increased ΔAICc; a ΔAICc of more than approximately 10 indicates almost no support for any other model than the best fit^87,90^. The number of regions in the best-fit model is given by the region score, calculated as the sum of potential numbers of regions modified by their Akaike weights; this value is equal to the hypothesised number of regions in the best-fit regionalization model. Uncertainty in the model is incorporated by this score. A model with a ΔAICc of more than approximately 10 will have a whole-number region score due to the robustness of the best-fit model. Region scores are expressed as the number of regions exclusive of the atlas-axis complex, which is shared by all tetrapods. In addition to the number of regions present, we analysed the degree of intracolumnar morphological heterogeneity for each taxon. We quantified heterogeneity as the mean Procrustes distance for all pairwise superimpositions within the vertebral column of each taxon. The Procrustes difference is a value representing the amount of difference between two sets of landmarks, or the degree of morphological difference between two objects. Along the presacral vertebral column of each specimen we calculated the Procrustes distance between each vertebra and every other vertebra using a double for-loop containing the procdist function in the shapes package in R (ver. 1.2.5)^91^. The output of this loop contains duplicate results, as the landmark set for each vertebra has been compared with every other set and every landmark set within its own set of comparisons. We subset a single, whole set of Procrustes distances using the unique function. We then took the mean of these Procrustes distances, which represents the mean difference in shape between every vertebra and every other vertebra, or the average amount of shape variability across the whole presacral vertebral column. We calculated heterogeneity in this way, rather than, for example, using the morphol.disparity function in *geomorph*, in order to standardise the method for taxa with varying numbers of vertebral elements. To ensure that this does not affect variation in the results we weighted this value by the number of vertebral elements under analysis to remove the effect whereby the mean intracolumnar heterogeneity calculated is lower for samples that encompass more vertebral elements. We multiplied each mean heterogeneity score by 100 for ease of comparison.

To test the impact of higher segment numbers on region modelling, we conducted analyses sampling the vertebral elements at 5% intervals^3^, so that all models were run on 20 or fewer elements, making no a-priori assumptions regarding anatomical transitions. For vertebral columns made up of fewer than 20 elements no change was made to the model. Where 5% intervals result in non-integer values intervals were rounded to the nearest whole number. For the majority of taxa, this resulted in no change to the number of recovered regions, and either no change to modelled region boundaries or shifts of one to two positions. For taxa that we originally modelled five or six regions (*Delma impar, Chalcides sepsoides, Chamaesaura anguina* and *Celestus hylaius*) 5% interval modelling reduced the resulting region scores by one to two units. We therefore chose to remove these taxa from further analysis, and did not sample additional taxa that have more than 40 presacral vertebrae, including snakes. Region and heterogeneity scores for 5% models are reported in Supplementary Data 1; region models and ancestral state reconstructions for region score and intracolumnar heterogeneity for 5% interval models are shown in Supplementary Fig. 8.

### Ancestral state estimation and evolutionary modelling

To model the evolutionary history of regionalization and heterogeneity, we performed ancestral state reconstructions on region numbers and heterogeneity values, using a temporally calibrated phylogeny for examined specimens. We calculated maximum likelihood ancestral states of intracolumnar heterogeneity using the contMap function in phytools. To reconstruct the evolutionary history of regionalization as a discrete quantity, as evolutionary changes between integer states, we conducted stochastic character mapping, using the make.simmap function in phytools. Despite region scores being calculated as continuous values, the biological property being quantified is the discrete number of regions, making continuous ancestral state reconstruction an inappropriate technique for this data. The state switches between 2, 3 and 4; An ancestral state of, for example, 3.42 regions is biologically meaningless. The make.simmap function first calculates the conditional likelihoods of each state at every node in the tree, given the branch lengths and the Q matrix of the chosen model, which represents the probability of changes between states. Then, using these likelihoods the algorithm simulates ancestral states at each node. Finally, the character history is derived using these simulated reconstructions and the data at the tips of the tree for each branch. The elements of the Q matrix used in the first stage of stochastic character mapping specify the probability of changes occurring between states. There are three possible models which have differently structured Q matrices: Equal rates (ER), the probability of each state change is the same; Symmetrical (SYM), the probability of state changes in one direction (e.g. from two regions to three regions, or from three regions to two regions) is the same; and All-rates-different (ARD), the probability of every state change is permitted to be different. We ran the analysis using all three models over 100 iterations, each time inputting the dated phylogeny and list of region scores rounded to the nearest integer, representing the discrete number of regions modelled for each taxon. The output of each model run includes the log(likelihood) of the model. The highest likelihood simmap output used the ARD model. However, we then used the following function to determine whether this increased likelihood is statistically significant:

3 $$p={{{\rm{pchisq}}}} \left[\right. 2 \,*\,[\log {Lik}({{{\rm{model}}}}1){\mbox{-}} \log {Lik}({{{\rm{model}}}}2)]$$

Where log*Lik* is the log likelihood of a given model output by make.simmap, the likelihood test statistic for the given pair of models, and pchisq is the chi-squared distribution function in R.

We conducted evolutionary modelling using the mvMORPH package in R^92^. For region scores and heterogeneity we fit nine different models: Passive (Brownian/random walk), Brownian with active trend, evolution towards a single evolutionary optimum (Ornstein-Uhlenbeck, OU), and six different multiple-optima OU models to test for shifting adaptive optima. We modelled Brownian motion using the mvBM function, and the OU models using mvOU. We fit the six multiple-optima OU models by manually creating partitions using the paintSubTree function in phytools – this enables the user to set clades as different groups, across which mvOU then models different adaptive optima. Four models were set to test different optima across each of these clades relative to the remainder of the tree: Archosauria, Lepidosauria, Pseudosuchia, Ornithodira, and the two remaining models were set to test different optima across two groups relative to the rest of the tree: Lepidosauria +  Archosauria, and Pseudosuchia + Ornithodira. We extracted AICc values for each model and calculated AIC weights to determine the best-fit model.

### Statistical testing

To test for differences in complexity metrics between ecological and taxonomic categories we conducted ANOVAs for region scores and intracolumnar heterogeneity, dividing taxa into: terrestrial, volant, fully aquatic, semi aquatic, fossorial and arboreal for ecology, and into the following clades: Amphibians, synapsids, birds, non-avian archosaurs and lepidosaurs (Supplementary Data 1). We conducted Tukey’s Honest Significant Difference (HSD) post-hoc tests to assess the significance of the differences in means between the categories, using the TukeyHSD function in R.

To model the relationships between axial skeletal heterogeneity, regionalization and body mass, we conducted linear regression using the lm function in R. To account for the effect of phylogenetic relationships on the relationship between body mass and heterogeneity we also conducted phylogenetic generalised least squares using the gls function in the nlme package^93,94^. This function enables the user to set parameters for the underlying evolutionary model. Ordinarily PGLS is conducted under the assumption of Brownian motion, but evolutionary modelling herein indicates that intracolumnar heterogeneity evolved under a multiple-optima OU model with a regime change across Archosauria. We used the corMartins function in ape^95^ to generate the correct correlation structure using the phylogeny and the alpha value modelled in mvMORPH for the best-fit model.

### Reporting summary

Further information on research design is available in the Nature Portfolio Reporting Summary linked to this article.

## Supplementary information

Supplementary Information

Description of Additional Supplementary Files

Supplementary Data 1

Supplementary Data 2

Reporting Summary

Transparent Peer Review file

## Source data

Source Data

## Data Availability

The landmark data generated in this study have been deposited in the github repository https://github.com/LucyEmmaRoberts/VertebralEvolution Meshes and CT data generated for this study have been deposited on Morphosource under accession code 000601963 https://www.morphosource.org/projects/000601963?locale=en Source data used to generate Figs. 1 and 2 are provided as a Source Data File. Data used to generate Figs. 3, 4 and 6 can be found in Supplementary Data 1. Data used to generate Fig. 5 is can be found in Supplementary Table 6. Source data are provided with this paper.

## Code availability

All code is available through public github repository https://github.com/LucyEmmaRoberts/VertebralEvolution.
