# Supplementary material for: Evolution of the reptile spine reveals independent trajectories to axial skeletal complexity in amniotes
Source: Nat Commun. 2026 Apr 16;17:5304. doi: 10.1038/s41467-026-72071-x (PMC13269487; doi:10.1038/s41467-026-72071-x)
Supplement: Supplementary file 1 — Supplementary Information [file 41467_2026_72071_MOESM1_ESM.pdf]

Supplementary Information:

Evolution of the reptile spine reveals independent trajectories to axial skeletal complexity in amniotes

Authors: L. E. Roberts[1,2]*, J. J. Head[1]

Affiliations:

[1]Department of Zoology and University Museum of Zoology, University of Cambridge; Cambridge, CB2 3EJ, UK

[2]Natural History Museum, London, SW7 5HD, UK

*Corresponding author Email: lucy.roberts@nhm.ac.uk

Supplementary Information includes:

Supplementary Methods
Supplementary Figures 1 to 12
Supplementary Tables 1 to 9

Supplementary Methods: Sensitivity Analysis
To decide the number and placement of landmarks, and to test the robustness of the landmarking and landmark processing procedures used in the regions analysis we designed a series of tests to determine the sensitivity to:

1. The number of landmarks and their placement
2. The number of principal components used mid-processing to determine the best-fit number of regions
3. Repeatability and intraspecific variability
4. Incomplete and/or damaged specimens

We carried out landmark reduction and principal component reduction tests on *Alligator mississippiensis*, *Prestosuchus chiniqueusis, Anolis carolinensis* and *Pelecanus erythrorhynchos*. This subsampled a wide taxonomic scope of the total sample, including presacral columns ranging from 18 vertebrae up to 24 vertebral elements, including both fossil and recent specimens.

1. Changing landmarks

We initially devised 17-landmarks to encompass all morphological extremities of each vertebra (Supplementary Fig. 2A). To test the effect of additional landmarks we added eight landmarks encompassing the lateral-most points of the centrum, the anterio-medial extent of the prezygapophyses and the posterior-medial extent of the postzygapophyses (Supplementary Fig. 2B), in order to encapsulate the length of zygapophyses rather than just the lateral extent in relation to the rest of the landmark constellation, and to capture the width of the centra rather than just the height. We then added eight additional landmarks to this 25-landmark model, at the medial and lateral meeting points of the centrum and neural arch, on the anterior and posterior faces of the vertebrae (Supplementary Fig. 2C). Archosaurs such as *Alligator mississippiensis* have a distinct connection between the neural arch and centrum whereas in squamates such as *Chamaeleo dilepis* this connection is less well-defined, making it difficult to consistently landmark the same homologous point across all vertebrae of all taxa. This 33-landmark test will gauge the impact of this observation.

In all other landmark reduction tests, we reduced the number of landmarks from the original 17. We performed four landmark reduction tests, two exploring the impact of removing landmarks encompassing the lateral extremes of vertebral morphology, and two exploring the impact of removing landmarks encompassing the medial and midline morphology. In lateral reduction test one (LR1, Supplementary Fig. 2D) we removed the landmarks on the diapophyses and parapophyses, leaving 13 remaining landmarks. In lateral reduction test two (LR2, Supplementary Fig. 2E) we took LR1 and also removed landmarks on the zygapophyses, leaving only nine landmarks along the midline. In medial reduction test one (MR1, Supplementary Fig. 2F) we removed all landmarks along the vertical midline except for those at the ventral extent of the centrum and the dorsal extent of the neural spine, leaving 12 landmarks. In medial reduction test two (MR2, Supplementary Fig. 2G) we took MR1 and removed the midline landmarks at the ventral and dorsal margins of the centrum and neural spine, leaving only eight landmarks on the zygapophyses and rib articulation surfaces.

To compare landmark tests between taxa we calculated the mean Procrustes distance in each analysis, a value signifying the degree of morphological heterogeneity between the presacral vertebrae of that specimen. We expected that models of more heterogeneous taxa would be more robust to landmark reduction than specimens with lower heterogeneity, due to the more subtle morphological gradients potentially present in less heterogeneous vertebral columns.

Changing Landmarks – results

The number of regions recovered in the best-fit model is robust to reduction in the number of landmarks in both tested extant archosaurs. All models for *Alligator mississippiensis* have four regions, and region boundaries shift only where lateral landmarks have been removed (Supplementary Fig. 3A). Even in the laterally reduced models shifted region boundaries differ only by one to two positions, and the modelled boundary between the third and fourth regions is identical in all tests. Regionalization patterns are identical for all models for *Pelecanus erythrorhynchos* except for the more extreme laterally reduced model (LR2) which retains only nine of the original landmarks along the medial plane of the vertebrae (Supplementary Fig. 3B). Even in this case only the boundary between regions one and two is shifted, and only by one vertebral position.

We recovered a four region best fit model in all landmark reduction tests for *Anolis carolinensis*, except for the more extreme midline reduction test (MR2), which reduces the number of modelled regions to three (Supplementary Fig. 3C). *Prestosuchus chiniqueusis* was the most sensitive tested specimen to reduced landmarks (Supplementary Fig. 3D). The first midline reduction test (MR1) maintains the same best fit model as the 33- 25- and 17-landmark analyses, whereas MR2

adds an additional region, splitting the third region into a third and fourth region. The first lateral reduction test (LR1) reduces the number of regions down to two but retains the same boundary position between regions one and two as in all models except for LR2, which has three regions with very different boundary positions to the three-region result modelled in the 33-, 25-, 17-landmark and MR1 tests.

Across all specimens the 33-landmark and 25-landmark sets produced identical best fit patterns of regionalization. The ∆AIC between the best fit model and the next-best fit model is similarly high in those derived from 33- and 25-landmark sets, except for the 33-landmark model for *Anolis carolinensis*, where the ∆AIC for the best fit 33-landmark model is 7.33 compared to 16.99 in the 25-landmark model. A ∆AIC of >10 generally indicates that the next-best fit model is a statistically implausible fit for the data but a ∆AIC of 7.33 is sufficiently high to consider rejection of the next best fit model[1,2,3].

Models derived from 17-landmark sets are identical to the 33- and 25-landmark models recovered for *Alligator mississippiensis, Prestosuchus chiniqueusis* and *Pelecanus erythrorhynchos*, and are similarly robust according to the ∆AIC between the chosen models and the next-best fit model. The 17-landmark model for *Anolis carolinensis* shifts the region boundaries between the second and third regions, and the third and furth regions, anterior-ward by one vertebra. In the first midline-reduction test (MR1) we modelled a regionalization pattern identical to the 33 and 25 landmark tests in all specimens, maintaining a ∆AIC indicating that all other models in that analysis can be rejected. In the second midline-reduction test (MR2) the same result is recovered for *Alligator mississippiensis* and *Pelecanus erythrorhynchos,* but the number of regions recovered in the best fit model in *Anolis carolinensis* and *Prestosuchus chiniqueusis* is altered. The first lateral-reduction test (LR1) had no effect on the best fit model for *P. erythrorhynchos.* LR1 altered the second region boundary by one position in *A. carolinensis*, and the first and second region boundaries by one position in the best fit model for *A. mississippiensis*. In the second lateral-reduction test (LR2) the boundary between regions one and two was shifted by one position in *P. erythrorhynchos*. In LR2 the first region boundary was shifted by another vertebra to the anterior in *A. mississippiensis* compared to LR1. Similarly, in *A. carolinensis* the first region boundary was shifted one position to the posterior compared to LR2. In *Prestosuchus chiniqueusis* LR2 produced a three-region best fit model.

The extant archosaurs *Alligator mississippiensis* and *Pelecanus erythrorhynchos* have the highest within-column heterogeneity of the taxa examined here (Supplementary Table 2), and are both unaffected by most reduction tests, and in lateral-reduction tests where modelled regions are affected, region boundaries are shifted by only one position. *Anolis carolinensis* is the least heterogenous of the four specimens (Supplementary Table 2) but the effect of landmark reduction has a more extreme and variable effect on region number and boundary positions modelled for *Prestosuchus chiniqueusis*. However, region boundaries modelled for *A. carolinensis* are affected at a greater number of landmarks, as the only specimen for which the 17-landmark model differs from the 33- and 25- landmark models. Heterogeneities calculated are also affected by the number of landmarks used. Lateral landmark reduction tests incurred large decreases in in mean Procrustes distances (Supplementary Table 2). Intracolumnar heterogeneity of *Prestosuchus chiniqueusis* is 62.43% lower when calculated using the LR2 landmarks when compared to the heterogeneity calculated using the original 17 landmarks. This implies that much less of the intracolumnar difference in shape along the vertebral column is encompassed by the midline of the vertebral elements compared to *A. mississippiensis*, *P. erythrorhynchos* or *A. carolinensis.* This result also demonstrates the importance of choosing a set of landmarks that comprehensively encompasses as much of the morphology of each vertebra as possible.

Changing landmarks – conclusions

Region models produced using specimens with highly heterogeneous, highly regionalized vertebral columns are remarkably robust to the reduction of landmarks. Test LR1 uses only 13 landmarks but when testing *Pelecanus erythrorhynchos* the same regions and a similarly robust ∆AIC are recovered as in all other tests except for LR2*.* Similarly, modelled regions are the same for all *Alligator mississippiensis* tests except for the two laterally reduced models. Even though these highly heterogeneous specimens can tolerate substantial landmark reduction, there is a greater impact on less heterogeneous taxa. The difference in modelled regions and their boundaries for *Anolis carolinensis* suggests that the 17-landmark plan may not be sufficient for modelling regionalization compared to the 33- and 25-landmark sets.

Increasing the number of landmarks used from 25 to 33 had no effect on the resulting region model for any specimen. The ∆AIC between the best fit model and the next best fit model is comparably robust between all 25- and 33-landmark analyses except for *Anolis carolinensis,* for which

the ∆AIC for the 33-landmark model is sufficient to consider rejection of the next best fit mode, whereas the ∆AIC for the 25-landmark region model is high enough such that any other model is statistically implausible. This reduction in robustness is slight but the potential for a loss in model strength using the 33-landmark model on less heterogeneous specimens implies that the 25-landmark model is more universally robust. Therefore, following the tests described here, we used the 25-landmark model (Supplementary Fig. 2B, Supplementary Table 1).

2. Changing Principal Components

Using the 25 landmark sets for each specimen, we ran the initial steps of the regions analysis (see Methods) using data for all principal components. We then chose a subset of this region data, discounting some of the SLR models for a selection of principal components. Continuing the regions analysis using this subset of region data we determined the maximum likelihood SLR model for each potential number of segments, and then determined which of these six models is the best-fit using the Akaike information criterion (AIC).

In this test we aimed to gauge the impact of reducing the number of principal components used, on the resulting best fit model and on the robustness of the result. Similar studies have conducted data reduction either choosing a standard number of principal components or by using functions built into the regions package to choose a number of principal components to remove[4]. Methods of principal component reduction include cutting off any that encompass less than a given proportion of variance, bootstrapping to remove components with eigenvalues less than the mean, or the function PCOmax[4] that determines the number of principal components to use that will maximize the resulting region score.

The bootstrapping technique can only be used on linear morphometric data, so here we tested the impact of reducing the number of principal components based on variance cutoffs and using the PCOmax function. We determined the relative robustness of resulting models by the change in ∆AIC between the best fit model and the next-best fit model. We ran this analysis using all principal components, using all principal components that encompass more than five percent of total morphological variation, all that encompass more than one percent, and those that contain more than 0.1% total variation. The total number of principal components differs for each specimen, equaling the number of vertebrae in the sample minus one. The number of principal components that fit within the above variance categories also varies between specimens. Test analyses range from using two principal components to all PCs; for *Anolis carolinensis* all of the principal components encompass more than 0.1% variation, so this test is identical to the original 25-landmark analysis. The PCOmax function calculates the number of principal components that will result in the greatest region score. For *Prestosuchus chiniqueusis* and *A. carolinensis* this is the same number of PCs as those that encompass > 5% total variation but is a unique number for all other taxa.

Changing PCs – results

Altering the number of principal components used in the region analysis had no impact on the resulting model for *Pelecanus erythrorhynchos* (Supplementary Fig. 4A), and all models are similarly robust, as indicated by ∆AICc values (Supplementary Table 3). Variation between models for *Anolis carolinensis* change very little, only the posterior-most region boundary changes, by a maximum of three positions (Supplementary Fig. 8B). For *Alligator mississippiensis* and *Prestosuchus chiniqueusis* reducing in the number of principal components altered the resulting model in some cases. All PC reduction tests for *Alligator mississippiensis* resulted in the same best-fit model except for the moderately reduced test, using five principal components, representing >1% total variation, and the PCOmax test, using four principal components. In contrast, for *Prestosuchus chiniqueusis* all tests resulted in the same model except for the most extreme reduction, using two principal components. The moderate reduction test here resulted in a reduced ∆AIC compared to the less-reduced models. The most reduced *A. mississippiensis* model, using just two principal components, representing >5% total variation, had a reduced ∆AIC of 4.77.

Changing PCs – conclusions

Reducing the number of principal components used to model regionalization alters the resulting number of regions and modelled region boundaries in unpredictable ways. Effects of PC reduction are variable across the four specimens; PC reduction does not have a linear or consistent effect on the output. For example, when reducing the number of PCs used to analyse regionalization in *Prestosuchus chiniqueusis* the number of modelled regions increases when using the fewest PCs (those encompassing >5% of total variation), whereas in the analyses of *Alligator mississippiensis* the

same model is produced when using the fewest PCs, all PCs and all PCs encompassing >0.1% variation, but the number of regions increases when using an intermediate number of PCs, all of those encompassing >1% of total variation. This is probably due to the reduction in data associated with using fossil specimens relative to specimens of extant taxa. Further data reduction by selecting fewer PCs may exacerbate the issue of reduced data availability. Similarly, ΔAIC varies in a non-linear fashion, sometimes increasing when the number of PCs is reduced and sometimes decreasing below the threshold at which the next-best fit model can be rejected.

This variability in the impact of reducing the proportion of principal components used suggests that care should be taken if data reduction is carried out. Reducing the amount of information input into the regions analysis alters the number of morphometric gradients that the regions analysis applies segmented linear regression to. This difference in morphometric variability between the total morphological variation and the amount of variation input into a regionalization analysis run using a reduced number of principal components may incur a loss of key information on how the vertebral column is regionalized. Removing principal components from the analysis follows an assumption that PCs encompassing less variation represent noise, but this assumes that more subtle morphological gradients are somehow not real components of morphometric difference. Geometric morphometrics is useful for analysis of regionalization because much of the intracolumnar variation is subtle. We find no benefit to such data reduction. Using all principal components output by the geometric morphometric analysis creates consistency in the method across separate analyses.

The PCOmax function either maintains the number of regions derived from using all principal components and maintains (*Anolis carolinensis*) or reduces the ΔAICc (*Pelecanus erythrorhynchos*); or increases the hypothesised number of regions (*Alligator mississippiensis*, *Prestosuchus chiniqueusis*). Artificially altering the amount of morphological information put into the regions analysis again assumes that more subtle intracolumnar morphological gradients are not useful or ‘true’ reflections of the morphology of that vertebral column. The theoretical basis or analytical benefit of using this function is dubious. Differences in the region scores calculated herein compared to Jones *et al.* 2018 likely reflect the difference in approach to PC reduction, alongside the difference in basing the models on 3D geometric data vs 2D linear data. We have taken the approach to make no prior assumptions regarding the amount of shape variation that represents ‘real’ biological signal or regarding key anatomical transitions, such that our analyses are not biased by prior assumptions. Therefore, throughout future analyses we used all principal components produced by geometric morphometric analyses, to avoid arbitrary decisions that evidently can have a marked impact on the resulting region score.

.

3. Intraspecific variability and repeatability

To gauge reproducibility of the region analysis methodology, and to test for intraspecific variability in regionalization, we processed and analysed CT scans of twelve specimens of *Sceloporus undulatus* using the 25-landmark plan (Supplementary Fig. 2B, Supplementary Table 1). Additionally, we processed and analysed two specimens of four crocodilian taxa: *Crocodylus niloticus, Tomistoma schlegelii, Crocodylus moreletti* and *Osteolaemus tetraspis*. Finally, we applied landmarks to copies of scans of the same specimen of *Chamaeleo chamaeleon* and *Gavialis gangeticus*, leaving several months between producing each repeated landmark set, to investigate the possibility of variation in the exact position of landmark coordinates induced by the collector.

Repeated analysis – results

Best fit models for all specimens of *Sceloporus undulatus* had four regions. The ΔAIC for each model was sufficiently high such that we were able to reject the next-best fit model. Modelling for only one specimen resulted in a ΔAIC < 10, but it was still sufficiently high to reject the next-best fit model. The boundaries between modelled regions varied between specimens. Across all twelve specimens the position of each boundary varied by two vertebrae (Supplementary Fig. 5). For example, the boundary between regions one and two lies between vertebrae five and six in eight out of the twelve analyses, for one specimen modelling recovered this boundary between vertebrae four and five, and for the remaining three specimens between vertebrae six and seven.

Similarly, duplicated analyses on different specimens of the same species of crocodilian produced models with the same number of regions. Boundary positions varied by one or two positions, except for *Crocodylus moreletti* for which both models are identical. Repeated analyses on the same specimen recovered identical region models (Supplementary Fig. 5) with similarly robust ΔAIC values to the original analyses (Supplementary Table 4).

Repeated analysis – conclusions

The number of regions is robust to intraspecific variation. Regional boundaries however vary between models produced using different specimens of the same species, by a limited extent but pervasively across the majority of tested taxa. Repeated analyses using the same specimen recover identical models, indicating that the differences in models made using different specimens of the same species are demonstrative of intraspecific variation rather than differences in landmark sampling.

4. Incomplete specimens

For effective geometric morphometric analysis all homologous edges, points and surfaces used must be accessible on all post atlas-axis vertebrae in the specimen. There are specimens, however, that do not fulfil this requirement, due to missing vertebral elements, damage (e.g. taphonomy) or because the vertebral column of a specimen is articulated, with centra and zygapophyses in contact. We carried out geometric morphometric analyses on damaged and incomplete specimens to test the limits of the methodology when the input data is incomplete. This will enable me to maximise the number of taxa included in my analysis, particularly of fossil specimens, which are more likely to be incomplete, and assure that specimens are removed that would not provide reliable results.

To test the impact of missing vertebral elements, we re-ran the regions analysis on a complete, undamaged specimen, *Alligator mississippiensis*, and simulated missing vertebrae by removing a whole matrix of coordinates from the TPS array of landmarks. Initially we ran regions analyses removing one vertebra, starting from the third and moving progressively through the vertebral column at regular intervals, running separate analyses removing vertebra 3, 7, 11, 15, 19 and 23. We originally attempted to run an analysis missing out the final vertebra, number 24, but the regions package does not allow for a missing element at the end, instead computing the analysis as if the presacral vertebral column terminates one vertebra earlier. The same issue did not arise when simulating the removal of vertebra three, the first post-atlas-axis vertebra, as 'regions' recognizes the presence of the atlas-axis complex without its inclusion in the landmark dataset.

Following these single removal analyses we increased the number of simulated missing vertebrae, taking each of the previous runs and removing an additional adjacent vertebra: removing 3 and 4, then 7 and 8, 15 and 16, 19 and 20, and 22 and 23. Following these runs we grouped double removals together, simulating specimens missing each pair of the groups of two vertebrae described above. We then extended this to run simulations missing three or four of the above groups of double vertebrae. Following this we re-ran the simulations on *Prestosuchus chiniqueusis* to gauge the effect of missing vertebrae on a less regionalized taxon; three regions are reconstructed in the presacral vertebral column of *P. chiniqueusis*, whereas four are recovered in *Alligator mississippiensis*. In addition to these analyses simulating missing vertebra, we re-examined the analyses of *Crocodylus acutus*. Vertebra 17 is missing from one of the two specimens. These specimens provide a non-simulated example of the effect of a missing vertebra.

Incomplete specimens – results

Recovered regions in *Alligator mississippiensis* maintain four regions in all simulations removing single vertebrae or two grouped vertebrae. Boundaries between regions were maintained in all cases except for the simulated removal of vertebra 11 and of vertebrae 11 plus 12, where the boundary between regions one and two shifts one position to the anterior, and the simulated removal of 15 plus 16, where the boundary between regions three and four shifts one position to the posterior. Analyses using both specimens of *Crocodylus acutus* recover four regions (Supplementary Fig. 5). Boundaries between regions one and two, and between two and three are shifted one position to the posterior in the specimen missing vertebra 17. The boundary between regions three and four is somewhat obscured in this specimen, as the missing vertebra is the final vertebra in region three in the complete specimen.

Resulting models of analyses in which two groups of double vertebrae (e.g. missing 3, 4, 11 and 12) are removed either retain the original four regions (Supplementary Fig. 6A) or breakdown to three regions (Supplementary Fig. 6B), depending both on how close together the two groups are and whether vertebrae 3 and 4 were missing from the analysis or not. When vertebrae 3 and 4 are missing, a three-region model is produced when the accompanying lost vertebrae are as close as 7 and 8 or 11 and 12, but the original four-region model is recovered when losing 3 and 4 is accompanied by the loss of vertebrae as distal as 15 and 16 or 19 and 20. However, when vertebrae 3 and 4 are present and two other groups of vertebrae (e.g. 7, 8, 11, 12) are missing the original, or one boundary shifted by one position, four-region model is recovered regardless of how close the two groups are. This same phenomenon is found when simulating losses of three groups of two

vertebrae. The original four region model is recovered only when one of the three groups is not vertebrae 3 and 4.

When vertebra three is missing, all simulated losses of multiple single vertebrae retained the original, or boundary shifted, four-region model until four individual vertebrae are lost, when the model breaks down to three regions. However, when running the same simulations including vertebra three, and instead removing vertebra four, the analysis was remarkably robust to additional losses. Running a simulation removing vertebrae 4, 7, 11, 15, 19, 21 and 23 maintains a four-region model. It is not until an additional vertebra, number 17, is removed that the resulting model is broken down to two regions. The specimen of *Prestosuchus chiniqueusis* was more sensitive to simulated losses than *Alligator mississippiensis* (Supplementary Fig. 6). All single-loss simulations maintained the original three-region result, but simulations removing two vertebrae resulted in a two-region model either when the losses include vertebra three or the missing vertebrae are close together, e.g., removing vertebrae 3 and 11 or 7 and 11 both result in two-region models. Any additional removals resulted in a two-region model.

Incomplete specimens – conclusions

The results of simulated missing vertebrae in *Alligator mississippiensis* suggest that otherwise undamaged, highly regionalized specimens of extant taxa are remarkably robust to missing vertebral elements. Regionalization analysis of less regionalized specimens, however, such as *Prestosuchus chiniqueusis* are more sensitive to missing more than one vertebra, but the original region models are retrieved where missing vertebrae are sufficiently far apart. This highlights a major concern for specimens with more than one missing vertebra: when analyzing a specimen that has more than one vertebra that is either missing or too damaged to landmark effectively it is not possible to discern how sufficiently far apart the missing elements should be for the results to be reliable. Therefore, one should be careful when using specimens that have missing vertebrae. These tests suggest that one missing vertebra is tolerable, but if multiple separate sections are missing, then careful consideration must be made to whether the specimen should be included in the analysis. Models are particularly sensitive to the absence of the first analysed vertebra, the third cervical (C3), when accompanied by additional missing vertebrae. Additionally, it is important to note that ∆AIC values cannot be used as a measure of whether a particular result has been derived from sufficient data. The ∆AIC value for a particular model gives the relative goodness of fit of that model relative to the other region models, all derived from the same data.

As a result of these findings, we removed several specimens from further analysis:

- *Brachychampsa montana*: 5/20 missing vertebrae, a group of three and a group of two separated by only one vertebra.
- *Dinornis maximus:* 11/27.
- *Gastornis sp.*: 3/21 missing vertebrae, including C3.
- *Haplocanthosaurus sp.*: 8/27 missing vertebrae, including C3.
- *Poposaurus gracilis:* 3/22 missing vertebrae, C3, 4, and 5.

Damaged specimens

In addition to missing elements, individual vertebrae may be incomplete or damaged. Vertebrae are series of similar and symmetrical structures; it is clearly apparent when an element is incomplete or damaged. As discussed above, it is imperative that specimens have as many useable vertebrae as possible. However, if a vertebral element was missing multiple parts, on both sides, or deformed such that it is not symmetrical, it could not reliably be used for geometric morphometric analysis, so we did not sample such vertebrae. If this results in multiple lost vertebrae then that whole specimen may no longer be useable, so it is important to ensure that as few vertebrae are removed as possible, to maximize the total number of specimens across the whole study. To ensure the greatest number of scans were compatible with geometric morphometric analysis we exploited the symmetrical form of vertebrae to digitally restore models, where possible. When a vertebral element was missing a structure on one side that was present on the other, we edited the model in Artec Studio (Supplementary Fig. 7). We edited the asymmetrical damaged meshes, produced a copy of the original mesh to the symmetrical structure from the undamaged side, mirrored it, and carefully placed it to make the model symmetrical, before merging with the original mesh (Supplementary Fig. 7). We conducted this symmetrical retrodeformation on the following elements: V14 of *Majungasaurus Crenatissimus* (missing postzygapophysis on one side), V14 of *Champsosaurus laramiensis* (missing prezygapophysis on one side), V5,6,7,8,16 of *Utahceratops gettyi* (missing transverse processes on one side), V21,22,23 of *Hesperornis regalis* (missing postzygapophysis on one side), V3,13,16,20 of

Procynosuchus (V13 missing postzygapophysis on one side, 3,16,20 missing prezygapophysis on one side), V5,8,9,10 *Stereosternum tumidum* (V8 missing postzygapophysis on one side, 5,9,10 missing prezygapophysis on one side), V21 *Anzu wyliei* (missing postzygapophysis on one side), and *Mus musculus* (V26 missing postzygapophysis on one side). Several elements of *Rutiodon sp.* and *Brachychampsa montana* were deformed such that they could not be reliably retrodeformed by this method, so we excluded these species from the analysis.

Phylogeny

Two of the taxa included herein, *Champsosaurus laramiensis* and *Dolichorhynchops osborni,* are members of clades that have uncertain phylogenetic positions within Reptilia. We tested the impact of changing phylogenetic positions on the ancestral state estimations. *C. laramiensis* is a choristodere. Choristodera has variably been recovered as a stem reptile or an archosauromorph[66-70]. *D. osborni* is a sauropterygian. Phylogenetic placement of Sauropterygia is similarly variable, either as ancestral to Lepidosauria + Archosauria, or as a stem archosaur[5,6,7]. In addition to the phylogenetic hypothesis used herein (Fig. 3, Supplementary Figure 1), we ran ancestral state estimations on phylogenetic hypotheses under the following scenarios:

A. Choristodera within Archosauromorpha; Sauropterigia as stem reptile
B. Choristodera as stem reptile; Sauropterigia within Archosauromorpha
C. Choristodera within Archosauromorpha; Sauropterigia within Archosauromorpha
D. Removing both *Champsosaurus laramiensis* and *Dolichorhynchops osborni*

We constructed each new topology and re-ran the tree dating procedure in paleotree (see methods), before running ancestral state estimation for region numbers and heterogeneity scores.

We recover no appreciable difference under any of these four alternative phylogenetic hypotheses (Supplementary Figure 11, Supplementary Table 9). Ancestral state probabilities for region number switch states only at the root node (Amniota + Lissamphibia). Under each of the above hypotheses, we model a three-region state instead of a two-region state. These alternative hypotheses however, do not change the highest probability ancestral state at the shared ancestor of amniotes, reptiles, lepidosaurs, archosauromorphs, pseudosuchians, ornithodirans, theropods or birds. If *Champsosaurus laramiensis* is placed as an archosauromorph, the probability of a three-region ancestral state the node uniting Archosauromorpha decreases to 68/69% versus 82-91%, and the probability of a two-region ancestral state increases in concert. Under every phylogenetic hypothesis tested, we maintain the same broad patterns of axial skeletal complexity across Amniota: The evolution of four regions from three-region ancestors in mammals, lepidosaurs and archosaurs, and a later loss of regionalization in theropods.

Supplementary References

1. K. P. Burnham, D. R. Anderson, K. P. Huyvaert, AIC model selection and multimodel inference in behavioral ecology: some background, observations, and comparisons. *Behav Ecol Sociobiol* 65, 23–35 (2011).

2. K. P. Burnham, D. R. Anderson, Multimodel Inference: Understanding AIC and BIC in Model Selection. *Sociological Methods & Research* 33, 261–304 (2004).

3. M. R. E. Symonds, A. Moussalli, A brief guide to model selection, multimodel inference and model averaging in behavioural ecology using Akaike's information criterion. *Behav Ecol Sociobiol* 65, 13–21 (2011).

4. Jones, K. E. et al. Fossils reveal the complex evolutionary history of the mammalian regionalized spine. *Science* 361, 1249–1252 (2018).

5. Neenan, J. M., Klein, N. & Scheyer, T. M. European origin of placodont marine reptiles and the evolution of crushing dentition in *Placodontia. Nat. Commun.* 4, 1621 (2013).

6. Schoch, R., Sues, HD. A Middle Triassic stem-turtle and the evolution of the turtle body plan. *Nature* 523 (2015): 584–587. https://doi.org/10.1038/nature14472

7. Simões, T.R., Kammerer, C.F., Caldwell, M.W. and Pierce, S.E. Successive climate crises in the deep past drove the early evolution and radiation of reptiles. *Science Advances* 8(33) (2022): eabq1898.

Supplementary Figures

Supplementary Figure 1. Phylogeny of taxa included in this study.

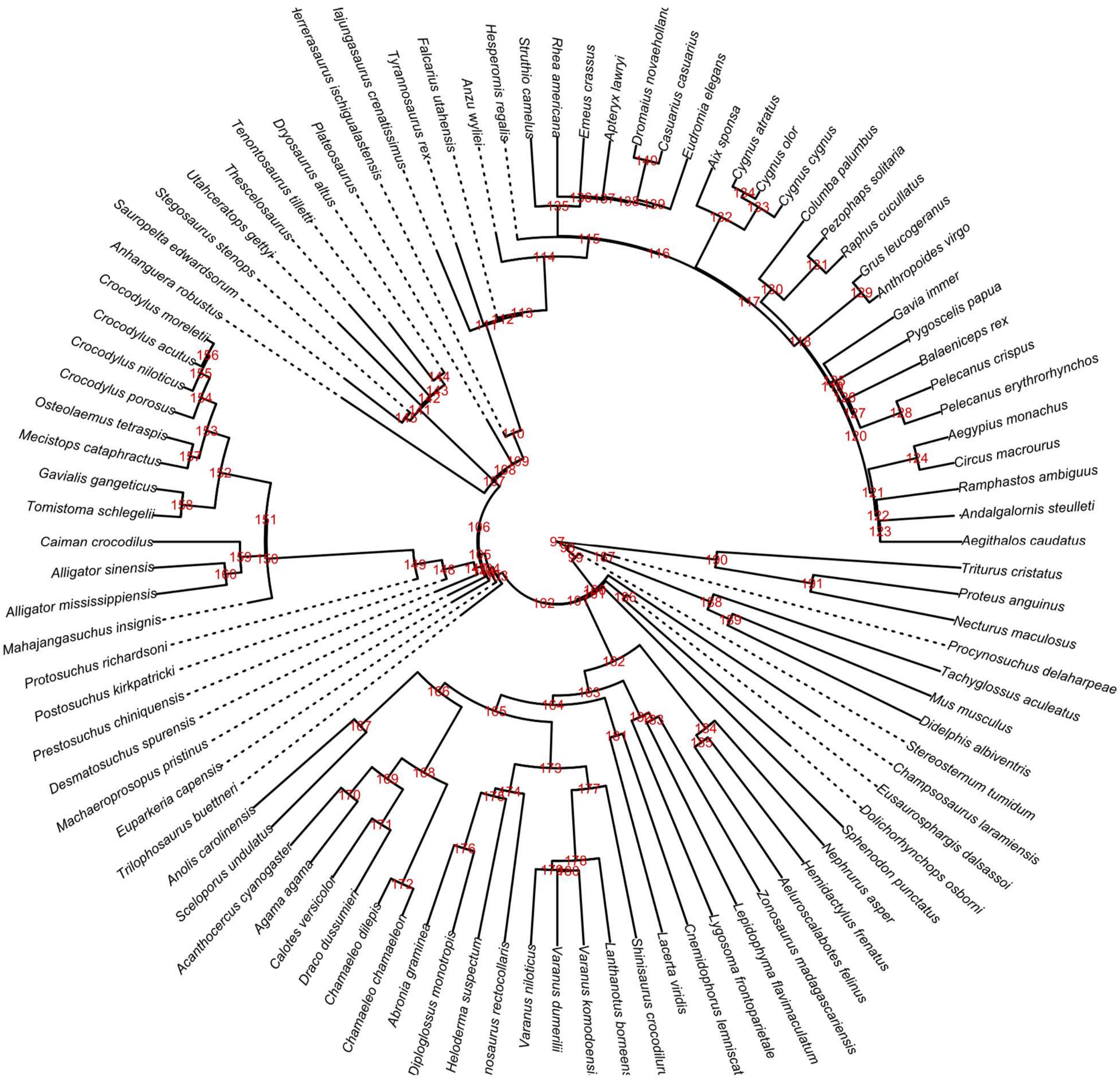

Supplementary Figure 2. Landmark configurations used in landmark sensitivity analysis, shown on the seventeenth presacral vertebra of *Alligator mississippiensis* TMM.M.4135 **A** 17 landmarks **B** 25 landmarks **C** 33 landmarks **D** Lateral reduction test one (LR1) **E** Lateral reduction test two (LR2) **F** Midline reduction test one (MR1) **G** Midline reduction test two (MR2)

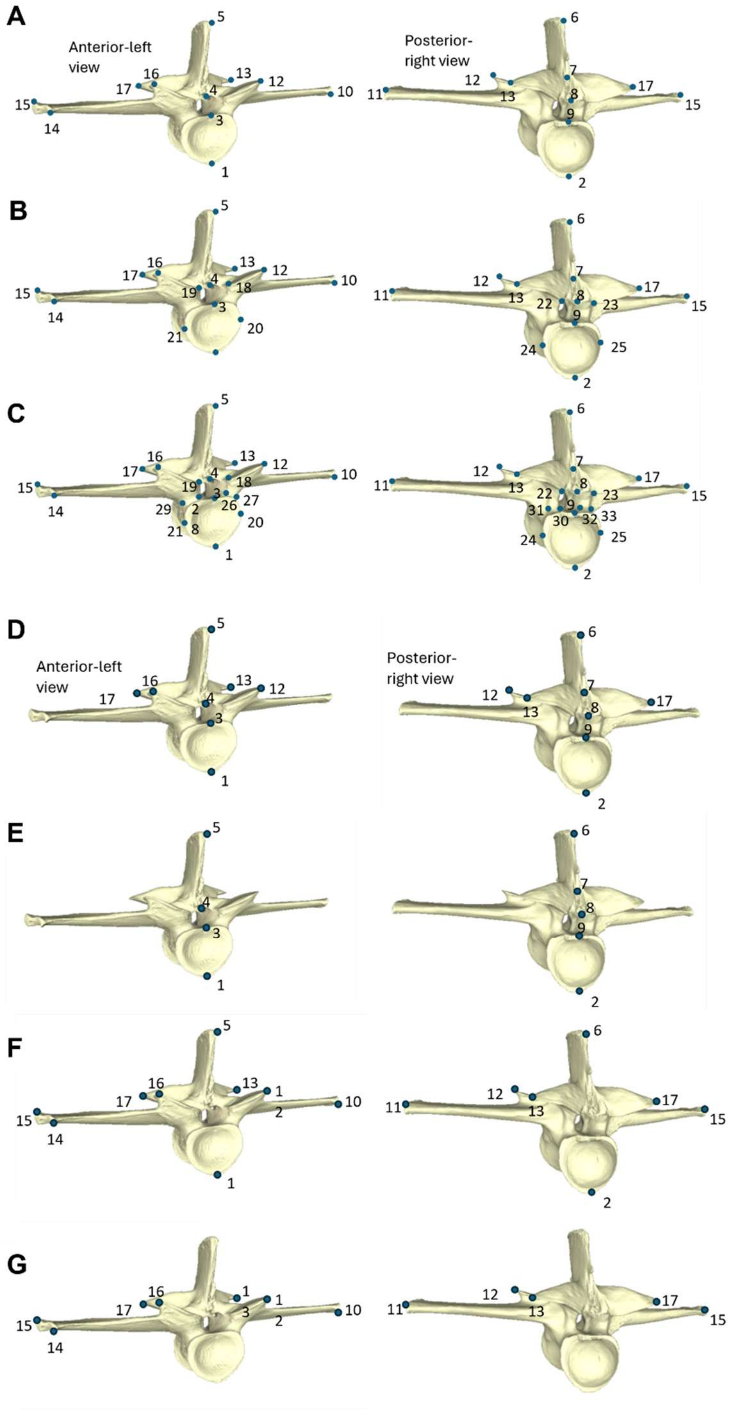

Supplementary Figure 3. Results of landmark reduction tests. Each region model is grouped by taxon and labelled with the underlying landmark configuration. **A** Results for *Alligator mississippiensis.* **B** Results for *Pelecanus erythrorhynchos.* **C** Results for *Anolis carolinensis.* **D** Results for *Prestosuchus chiniqueusis.*

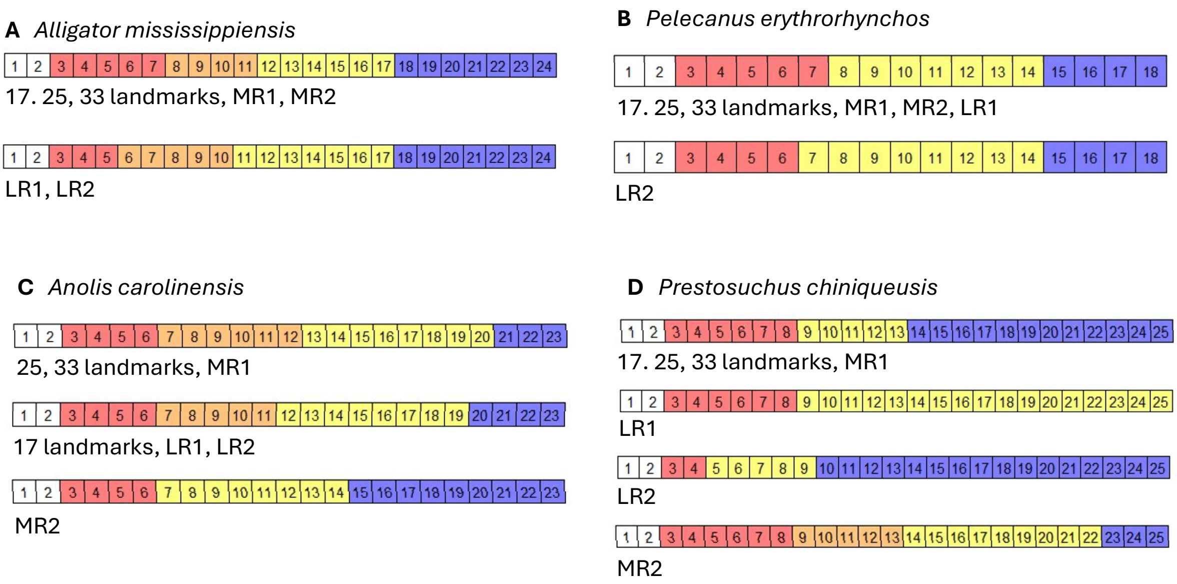

Supplementary Figure 4. Results of principal component reduction tests. Each region model is grouped by taxon and labelled to indicate the proportion of variance encompassed by the input principal component data, e.g. >5% VAR indicates that model was produced using all principal components that encompass more than %5 of the total variation. **A** *Pelecanus erythrorhynchos.* **B** *Anolis carolinensis.* **C** *Alligator mississippiensis.* **D** *Prestosuchus chiniqueusis.*

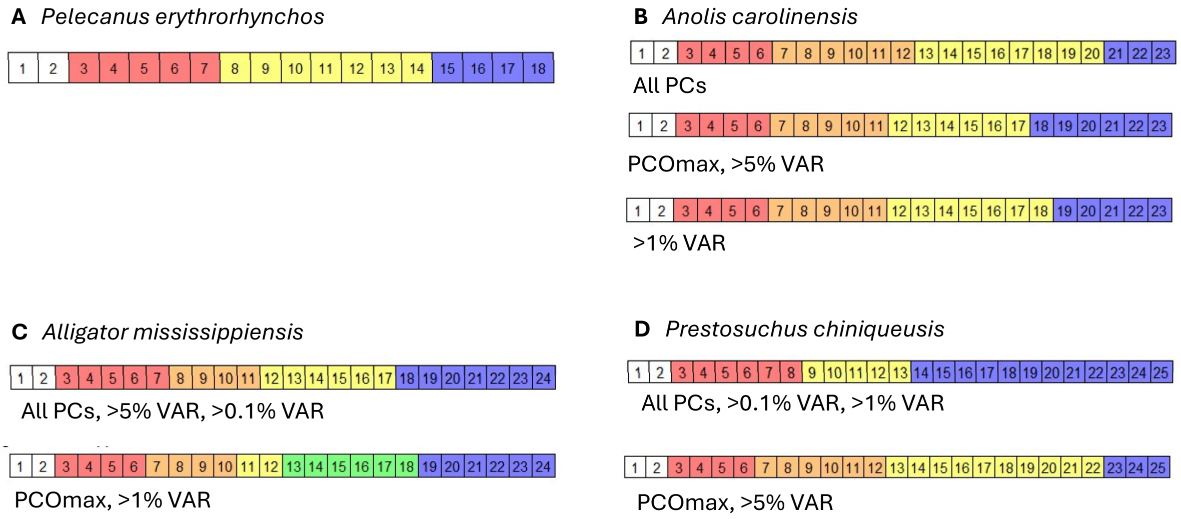

Supplementary Figure 5. Results of duplicate analyses on multiple specimens of the same taxon. Split colour boxes represent vertebral elements that vary in regional identity between specimens.

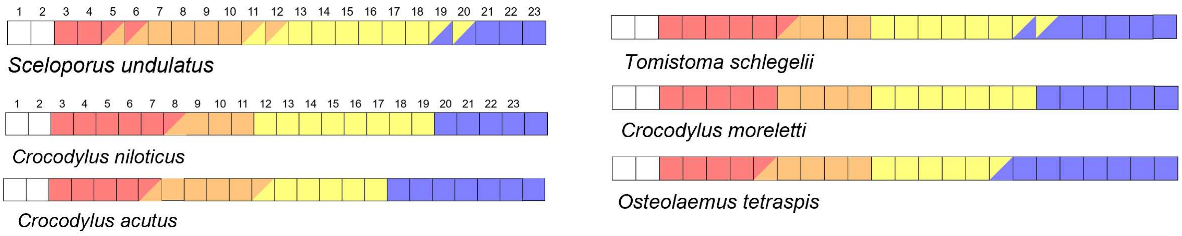

Supplementary Figure 6. Region models for with simulated missing vertebrae **A** *Alligator mississippiensis* models retaining four regions **B** *Alligator mississippiensis* models which lose (a) region(s) **C** *Prestosuchus chiniqueusis* models retaining four regions **D** *Prestosuchus chiniqueusis* models which lose (a) region(s).

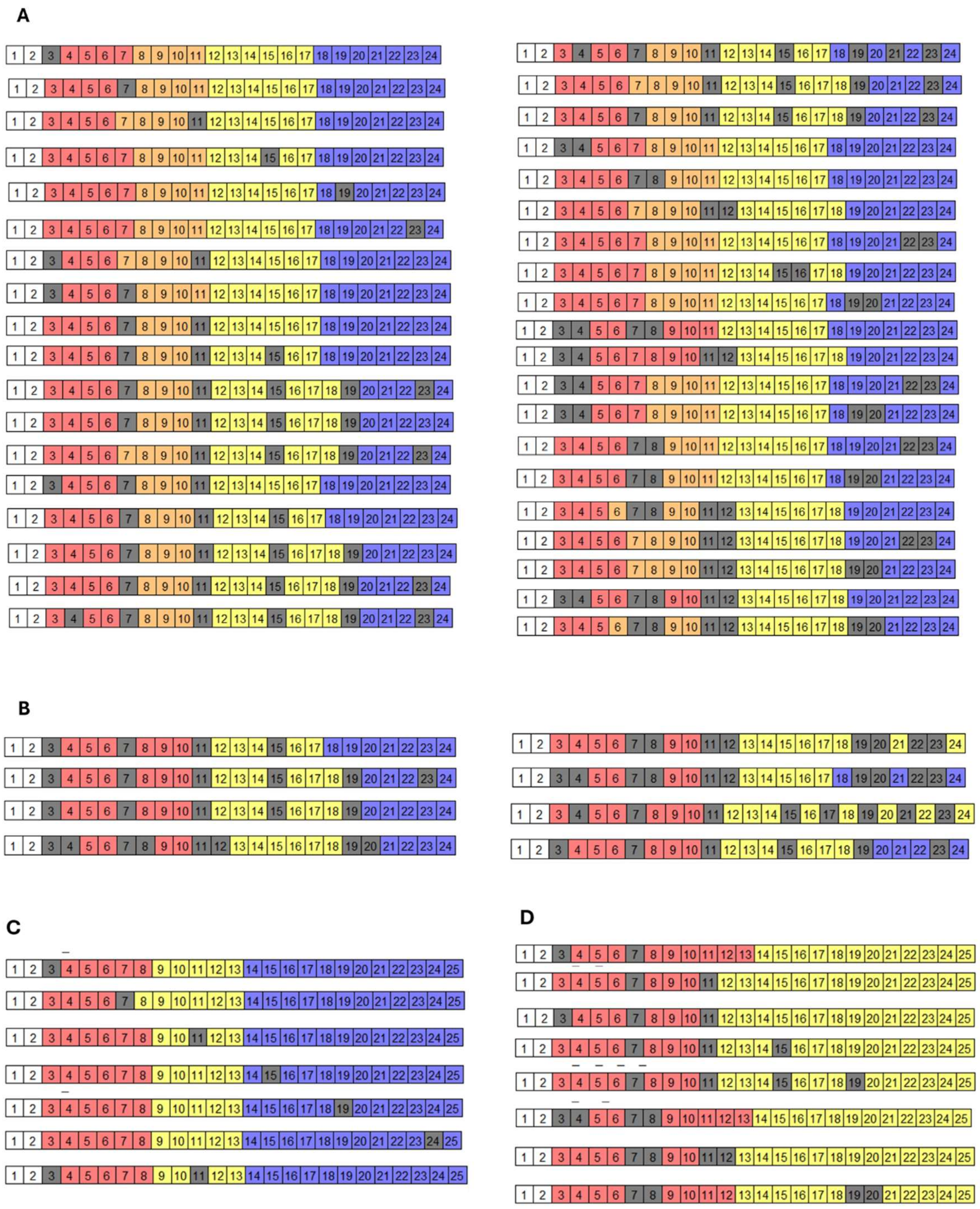

Supplementary Figure 7. Vertebra 18 of *Gastornis gigantea* FMNH.PA.34. **1**: original mesh with missing transverse process **2**: a copy of the original mesh **3**: the copy reduced to the portion missing from the right-hand side (transverse process) **4**: mirrored transverse process **5**: transverse process placed to make the mesh symmetrical **6**: Final mesh for landmarking, formed by merging the original mesh with the mirrored transverse process.

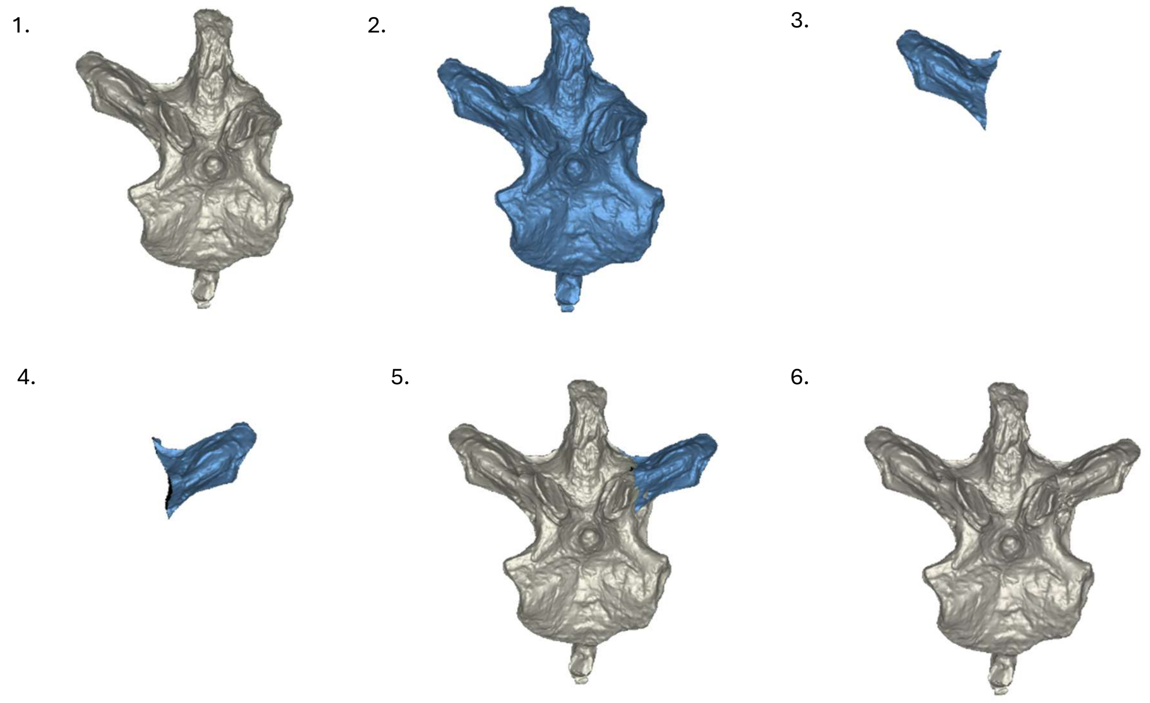

Supplementary Figure 8. Modelled number of regions, intracolumnar heterogeneity and regionalization models across the sample for dataset sampling 5% intervals (see Supplementary Methods). Silhouettes were accessed via PhyloPic and have all been dedicated to the public domain (CC0 1.0 Universal Public Domain Dedication Licence).

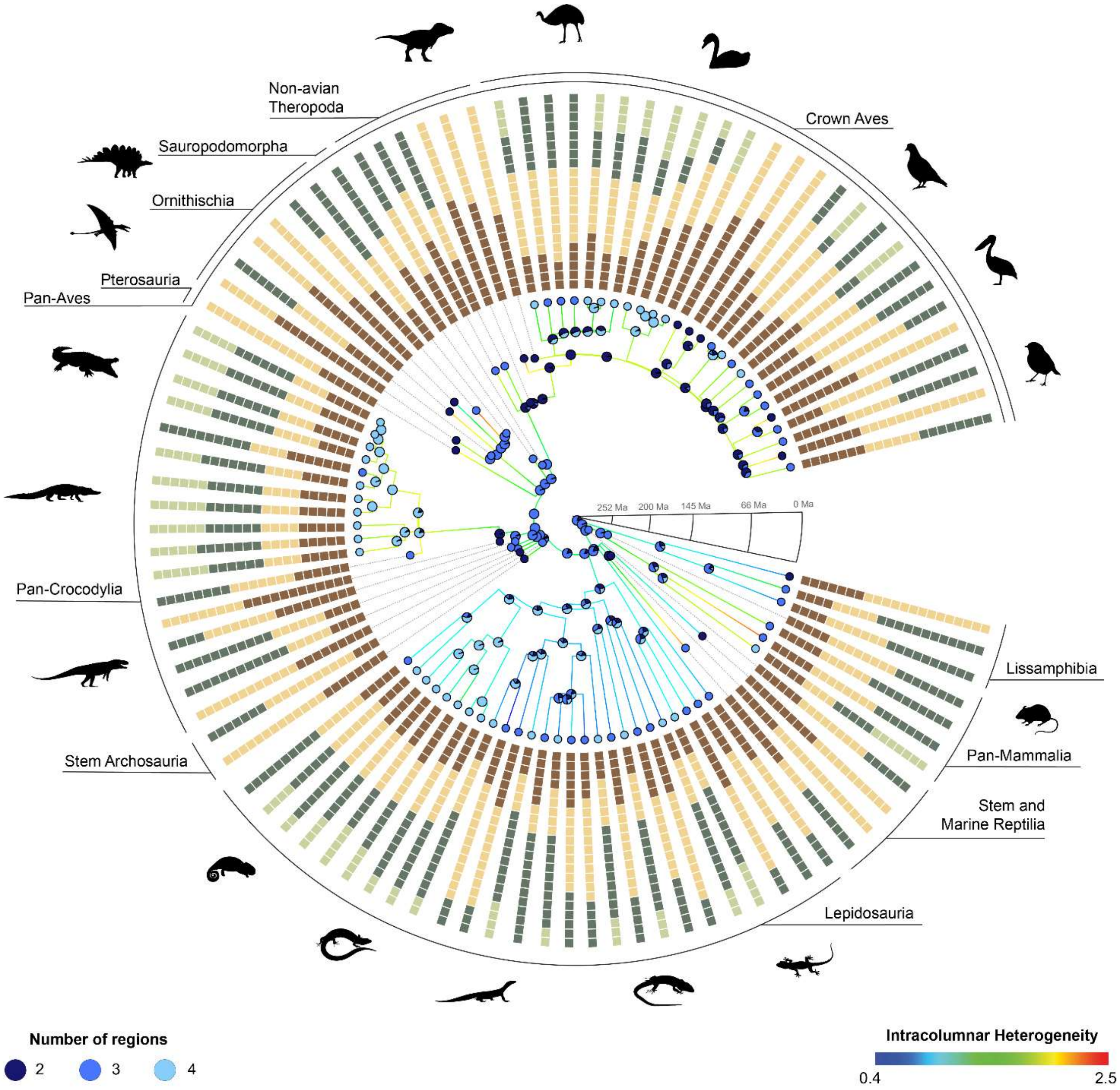

Supplementary Figure 9. Regionalization and vertebral morphology across Reptilia. Colours represent modelled regional identity. **A** Lepidosauria: **i** *Chamaeleo chamaeleon* ii *Nephrurus asper* **B** Theropoda: **i** *Tyrannosaurus rex* **ii** *Anzu wyliei* **iii** *Cygnus atratus* **iv** *Ramphastos ambiguus*. C Pan-Crocodylia: **i** *Alligator mississippiensis* **ii** *Mahajangasuchus insignis.*

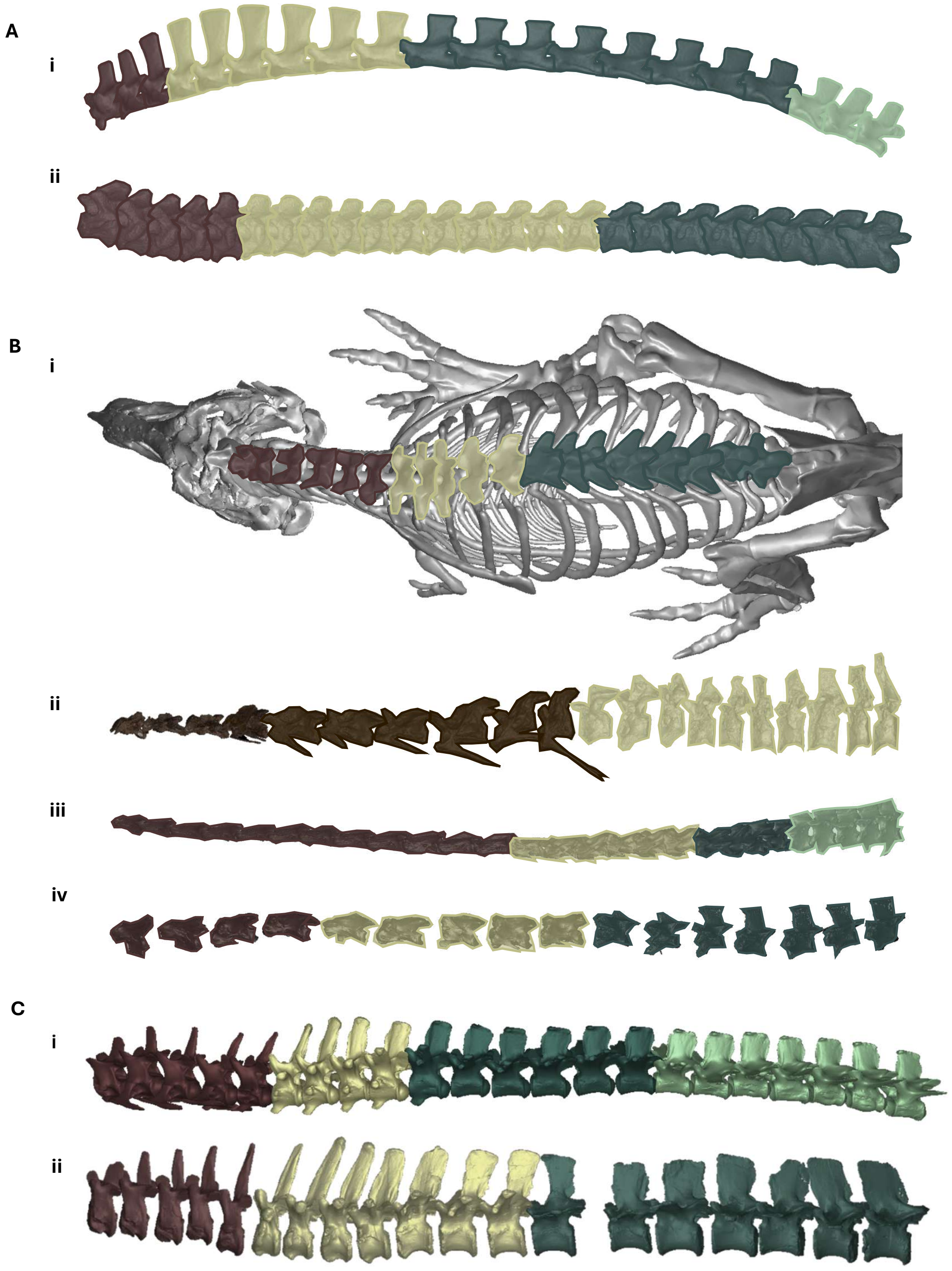

Supplementary Figure 10. Maximum likelihood ancestral state maps of residual values of heterogeneity following least squares regression with body mass, and unaltered heterogeneity scores, across the whole sample.

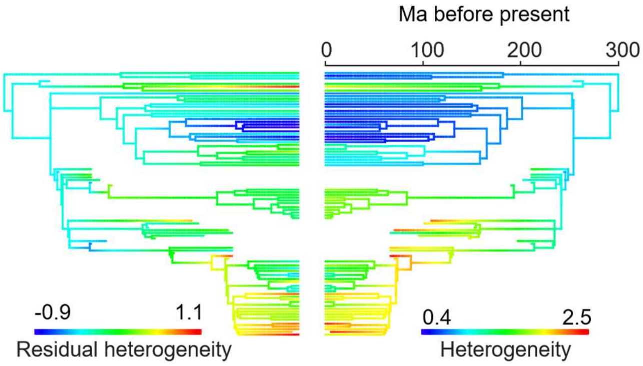

Supplementary Figure 11. Ancestral state estimations of heterogeneity and region number for alternative phylogenetic hypotheses. **A.** *Champsosaurus laramiensis* as a stem archosaur, *Dolichorhynchops osborni* as a stem reptile. **B.** *Champsosaurus laramiensis* as a stem reptile, *Dolichorhynchops osborni* as a stem archosaur. **C.** *Champsosaurus laramiensis* as a stem archosaur, *Dolichorhynchops osborni* as a stem archosaur. **D.** Both taxa removed.

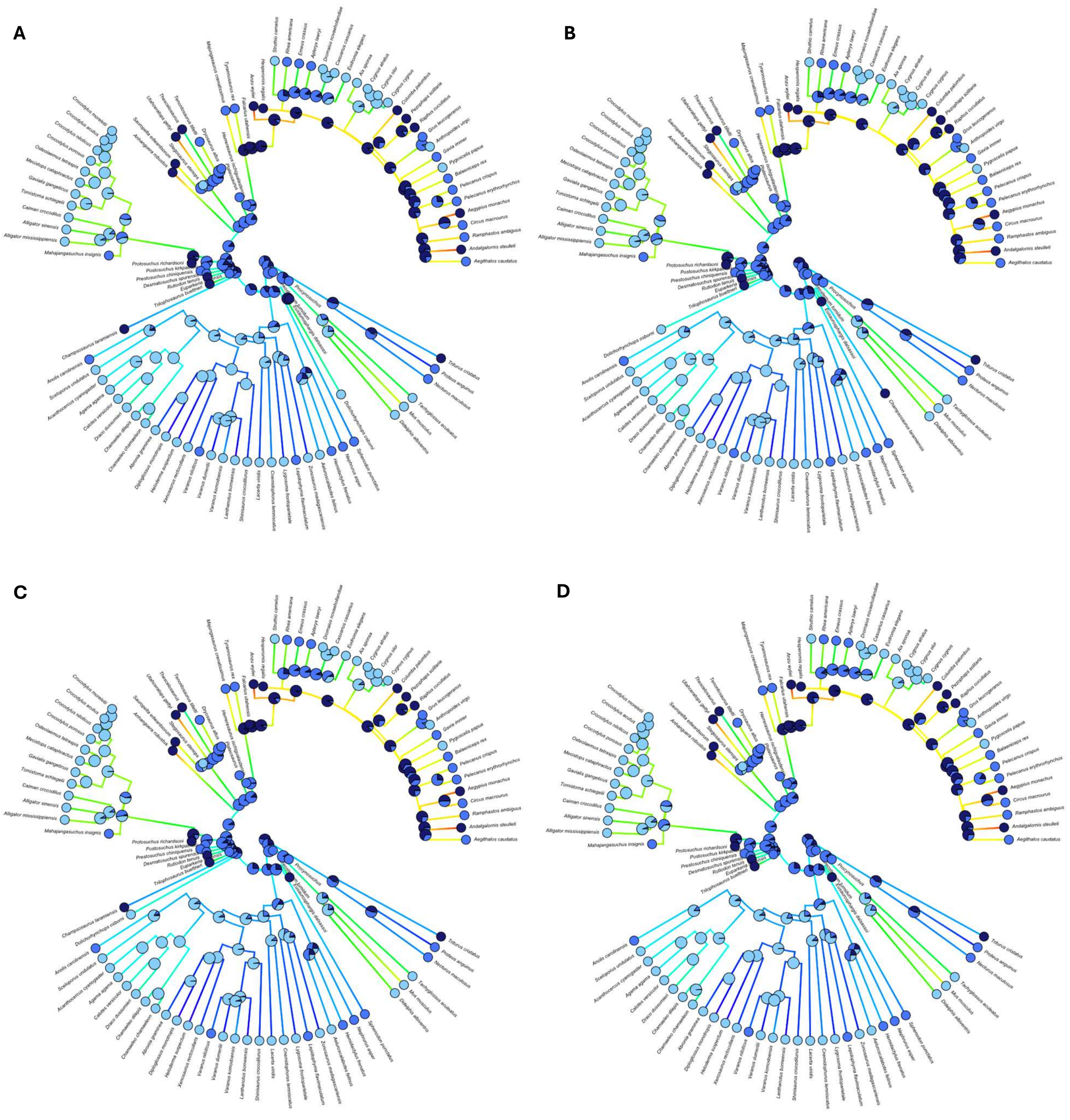

Supplementary Figure 12. Regression of body mass against region score.

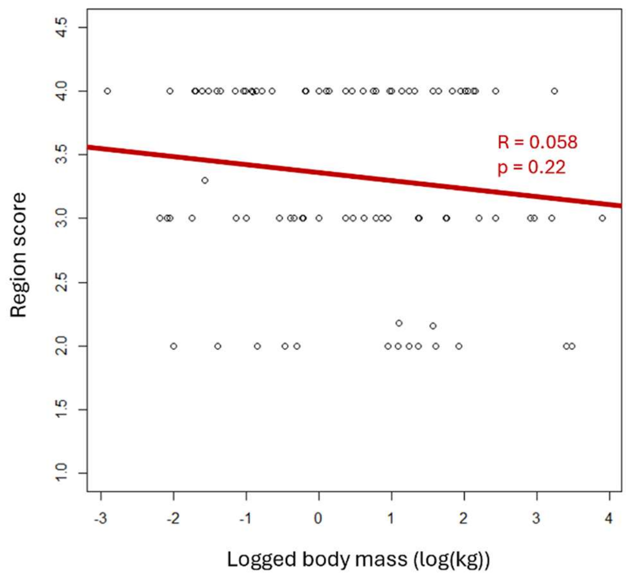

Supplementary Tables

Supplementary Table 1. Description of the landmarks used in regionalization analyses.

Landmark number	Description
**1**	Anterior ventral midline of centrum
**2**	Posterior ventral midline of centrum
**3**	Anterior dorsal midline of centrum
**4**	Anterior ventral midline of neural arch
**5**	Anterior dorsal midline of neural arch, midline articulation with neural spine
**6**	Posterior dorsal midline of neural spine
**7**	Posterior ventral midline of neural spine
**8**	Posterior midline of neural arch, midline articulation with neural spine
**9**	Posterior dorsal midline of centrum
**10**	Anteroventral edge of right-hand parapophysis/transverse process/apophysis
**11**	Posterodorsal edge of right-hand diapophysis/transverse process/apophysis
**12**	Lateral-most point of the anterior edge of right-hand prezygapophysis
**13**	Lateral-most point of the posterior edge of right-hand postzygapophysis
**14**	Posterodorsal edge of left-hand parapophysis/transverse process/apophysis
**15**	Dorsolateral edge of left-hand diapophysis/transverse process/apophysis
**16**	Lateral-most point of the anterior edge of left-hand prezygapophysis
**17**	Lateral-most point of the posterior edge of left-hand postzygapophysis
**18**	Anteromedial point of the articulation surface between the right-hand prezygapophysis and the neural arch
**19**	Anteromedial point of the articulation surface between the left-hand prezygapophysis and the neural arch
**20**	Right-lateral extent of anterior centrum articulation
**21**	Left-lateral extent of anterior centrum articulation
**22**	Posteromedial point of the articulation surface between the right-hand postzygapophysis and the neural arch
**23**	Posteromedial point of the articulation surface between the left-hand postzygapophysis and the neural arch
**24**	Right-lateral extent of posterior centrum articulation
**25**	Left-lateral extent of posterior centrum articulation

Supplementary Table 2. Statistics associated with sensitivity analyses run using different landmark numbers.

Taxa and landmark test	Region score	ΔAICc	Heterogeneity
Alligator mississippiensis 33	4.00	103.32	0.380
Alligator mississippiensis 25	4.00	76.17	0.385
Alligator mississippiensis 17	4.00	65.07	0.400
Alligator mississippiensis MR1	4.00	39.04	0.399
Alligator mississippiensis MR2	4.00	56.91	0.407
Alligator mississippiensis LR1	4.00	97.84	0.230
Alligator mississippiensis LR2	4.00	69.95	0.189
Pelecanus erythrorhynchos 33	3.00	77.21	0.308
Pelecanus erythrorhynchos 25	3.00	71.05	0.303
Pelecanus erythrorhynchos 17	3.00	58.42	0.335
Pelecanus erythrorhynchos MR1	3.00	46.58	0.349
Pelecanus erythrorhynchos MR2	3.00	47.34	0.330
Pelecanus erythrorhynchos LR1	3.00	53.45	0.271
Pelecanus erythrorhynchos LR2	3.00	49.61	0.279
Anolis carolinensis 33	3.97	7.33	0.201
Anolis carolinensis 25	4.00	16.99	0.198
Anolis carolinensis 17	4.00	32.56	0.196
Anolis carolinensis MR1	4.00	43.80	0.189
Anolis carolinensis MR2	3.02	7.60	0.187
Anolis carolinensis LR1	4.00	94.33	0.176
Anolis carolinensis LR2	4.00	75.33	0.168
Prestosuchus chiniqueusis 33	2.99	10.57	0.256
Prestosuchus chiniqueusis 25	3.00	20.82	0.252
Prestosuchus chiniqueusis 17	3.00	37.05	0.267
Prestosuchus chiniqueusis MR1	3.00	18.46	0.260
Prestosuchus chiniqueusis MR2	3.81	2.88	0.313
Prestosuchus chiniqueusis LR1	2.01	8.62	0.144
Prestosuchus chiniqueusis LR2	3.00	11.70	0.100

Supplementary Table 3. Statistics associated with sensitivity analyses run using different proportions of the principal component data.

Taxon and principal component test	# PCs	Region score	ΔAIC
***Alligator mississippiensis* - all PCs**	**21**	**4.00**	**76.17**
Alligator mississippiensis PCs > 5% var	2	4.08	4.77
Alligator mississippiensis PCs > 1% var	5	4.99	18.47
Alligator mississippiensis PCs > 0.1% var	11	4.00	31.02
Alligator mississippiensis PCOmax	4	5.00	15.79
***Prestosuchus chiniqueusis* - all PCs**	**22**	**3.00**	**20.82**
Prestosuchus chiniqueusis PCs > 5% var	2	4.00	17.96
Prestosuchus chiniqueusis PCs > 1% var	6	3.12	3.93
Prestosuchus chiniqueusis PCs > 0.1% var	20	3.00	20.05
Prestosuchus chiniqueusis PCOmax	2	4.00	17.96
***Pelecanus erythrorhynchos* - all PCs**	**15**	**3.00**	**71.05**
Pelecanus erythrorhynchos PCs > 5% var	3	3.00	23.26
Pelecanus erythrorhynchos PCs > 1% var	6	3.00	30.21
Pelecanus erythrorhynchos PCs > 0.1% var	14	3.00	66.89
Pelecanus erythrorhynchos PCOmax	4	3.00	17.84
***Anolis carolinensis* - all PCs**	**20**	**4.00**	**16.99**
Anolis carolinensis PCs > 5% var	3	4.00	18.29
Anolis carolinensis PCs > 1% var	6	4.00	24.96
Anolis carolinensis PCs > 0.1% var	20	4.00	16.99
Anolis carolinensis PCOmax	3	4.00	18.29

Supplementary Table 4. Statistics associated with sensitivity analyses run on different specimens of the same species

Taxon	Specimen	Regions score	ΔAIC	Heterogeneity
Sceloporus undulatus	1	4.00	39.32	0.214
Sceloporus undulatus	2	4.00	40.64	0.215
Sceloporus undulatus	3	4.00	56.17	0.207
Sceloporus undulatus	4	4.00	26.38	0.203
Sceloporus undulatus	5	4.00	20.67	0.222
Sceloporus undulatus	6	4.00	24.43	0.218
Sceloporus undulatus	7	4.00	36.78	0.210
Sceloporus undulatus	8	4.00	11.56	0.195
Sceloporus undulatus	9	4.00	99.88	0.207
Sceloporus undulatus	10	3.99	8.62	0.213
Sceloporus undulatus	11	4.00	41.86	0.207
Sceloporus undulatus	12	4.00	45.94	0.208
Crocodylus niloticus	UMZC	4.00	70.06	0.377
Crocodylus niloticus	RVC	4.00	41.40	0.356
Crocodylus acutus	UMZC	4.00	30.90	0.388
Crocodylus acutus	TMM	4.00	76.30	0.354
Crocodylus moreletti	UMZC	4.00	16.45	0.405
Crocodylus moreletti	TMM	4.00	116.8	0.380
Tomistoma Schlegelii	UMZC	4.00	71.56	0.394
Tomistoma Schlegelii	TMM	4.00	48.66	0.367
Osteolaemus tetraspis	RVC_FDC1	4.00	59.36	0.350
Osteolaemus tetraspis	RVC_FDC2	4.00	93.95	0.374
Chamaeleo chamaeleon	UMZC	4.00	80.46	0.249
Chamaeleo chamaeleon	UMZC	4.00	103.15	0.251
Gavialis gangeticus	UMZC R.5783	4.00	102.67	0.383
Gavialis gangeticus	UMZC R.5783	4.00	91.11	0.381

Supplementary Table 5. Topologies used to construct phylogenetic hypotheses used in this study.

Clade	Phylogeny Reference
Choristodera	Evans 1988; Gao & Fox 1998; Dudgeon *et al.* 2020; Jiang *et al.* 2023; Guillaume *et al.* 2025
Sauropterygia	Neenan *et al.* 2013; Schloch & Sues 2015; Simões *et al.* 2022
Lepidosauria	Zheng and Wiens 2015
Stem Archosauria	Sookias 2016; Nesbitt *et al.* 2013
Pan-Crocodylia	Wu *et al.* 2001; Bronzati *et al.* 2012; Desojo *et al.* 2020
Pterosauria	Britt *et al.* 2018
Ornithopoda	Prieto-Marquez 2010; McDonald 2012, Herne *et al.* 2019; Dieudonné *et al.* 2021
Thyreophora	Thompson *et al.* 2012
Sauropoda	Müller 2020
Theropoda	Hendrickx *et al.* 2015; TimeTree; Prum *et al.* 2015

Supplementary Table 6. Probabilities of region scores at each node in the phylogeny modelled using stochastic character mapping under an all-rates-different model (numbered according to Supplementary Fig. 1).

Node	2	3	4
**97**	0.58	0.42	0
**98**	0.3	0.7	0
**99**	0.1	0.9	0
**100**	0.3	0.7	0
**101**	0.27	0.73	0
**102**	0.24	0.76	0
**103**	0.15	0.85	0
**104**	0.08	0.92	0
**105**	0.23	0.77	0
**106**	0.24	0.76	0
**107**	0.29	0.71	0
**108**	0.3	0.7	0
**109**	0.29	0.71	0
**110**	0.1	0.9	0
**111**	0.87	0.13	0
**112**	0.92	0.08	0
**113**	0.99	0.01	0
**114**	0.61	0.39	0
**115**	0.85	0.15	0
**116**	0.81	0.19	0
**117**	0.76	0.24	0
**118**	0.51	0.49	0
**119**	0.48	0.52	0
**120**	0.46	0.54	0
**121**	0.44	0.56	0
**122**	0.45	0.55	0
**123**	0.45	0.55	0
**124**	0.43	0.57	0
**125**	0.41	0.59	0
**126**	0.39	0.61	0
**127**	0.34	0.66	0
**128**	0.26	0.74	0
**129**	0.17	0.72	0.11
**130**	0.41	0.59	0
**131**	0.58	0.42	0
**132**	0.01	0.02	0.97
**133**	0	0	1
**134**	0	0	1
**135**	0.45	0.53	0.02
**136**	0.35	0.64	0.01
**137**	0.33	0.66	0.01
**138**	0.32	0.66	0.02
**139**	0.2	0.44	0.36
**140**	0	0	1
**141**	0.19	0.8	0.01
**142**	0.17	0.83	0
**143**	0.16	0.84	0
**144**	0.06	0.94	0
**145**	0.13	0.62	0.25
**146**	0.24	0.76	0
**147**	0.26	0.74	0
**148**	0.15	0.85	0
**149**	0.88	0.12	0
**150**	0.12	0.37	0.51
**151**	0.11	0.27	0.62
**152**	0.01	0.01	0.98
**153**	0	0	1
**154**	0	0	1
**155**	0	0	1
**Node**	**2**	**3**	**4**
**156**	0	0	1
**157**	0	0	1
**158**	0	0	1
**159**	0.01	0.04	0.95
**160**	0.01	0.02	0.97
**161**	0.24	0.76	0
**162**	0.23	0.37	0.4
**163**	0.07	0.21	0.72
**164**	0.05	0.08	0.87
**165**	0.02	0.03	0.95
**166**	0.03	0.01	0.96
**167**	0.05	0.14	0.81
**168**	0	0	1
**169**	0	0	1
**170**	0	0	1
**171**	0	0	1
**172**	0	0	1
**173**	0	0	1
**174**	0	0	1
**175**	0	0	1
**176**	0	0	1
**177**	0	0	1
**178**	0	0	1
**179**	0	0.01	0.99
**180**	0	0.01	0.99
**181**	0.07	0.02	0.91
**182**	0.06	0.19	0.75
**183**	0.08	0.17	0.75
**184**	0.27	0.45	0.28
**185**	0.31	0.41	0.28
**186**	0.88	0.12	0
**187**	0.19	0.81	0
**188**	0.13	0.16	0.71
**189**	0.13	0.15	0.72
**190**	0.27	0.71	0.02
**191**	0.42	0.53	0.05

Supplementary Table 7. Results of evolutionary modelling for region scores, heterogeneity, and (log) body mass within each clade, best-fit model weights for each trait are highlighted in grey.

Evolutionary model	Region score: AICc	Region score: AIC weights	Heterogeneity: AICc	Heterogeneity: AIC weights	Log body mass: AICc	Log body mass: AIC weights
Brownian Motion (BM)	226.36	0	-821.07	0	301.9	0
BM with trend	225.41	0	-811.7	0	303.7	0
Ornstein-Uhlenbeck (OU) single optimum	200.43	0.02	-813.75	0	298.48	0
OU optima shift: Pan-Archosauria	198.95	0.05	-838.07	0.76	283.25	0.65
OU optima shift: Lepidosauria	194.12	0.54	-834.77	0.15	295.31	0
Multiple OU optima shifts: Pan-Crocodylia & Pan-Aves	198.79	0.05	-833.61	0.08	286.47	0.13
Multiple OU optima shifts: Pan-Archosauria & Lepidosauria	196.39	0.18	-829.31	0.01	285.55	0.21
OU optima shift: Pan-Aves	196.79	0.14	-829	0.01	292.1	0.01
OU optima shift: Pan-Crocodylia	201.51	0.01	-814.07	0	299.5	0

Supplementary Table 8. Distribution of region scores and intracolumnar heterogeneity by taxonomic group and ecological category. Significant results ($p < 0.05$) are highlighted in green. Differences among groups were analysed using one-way ANOVA (two-sided), followed by Tukey's honest significant difference (HSD) test for multiple comparisons with family-wise error adjustment.

**Region scores by clade**	**diff**	**lwr**	**upr**	**p**
Lepidosauria - Crown Aves	0.626538	0.091271	1.161806	0.013397
Lissamphibia - Crown Aves	-0.48718	-1.66396	0.689597	0.777742
Pan-Mammalia - Crown Aves	0.596154	-0.44039	1.632694	0.500247
Pan-Archosauria (excluding Crown Aves) - Crown Aves	-0.08502	-0.58782	0.417773	0.989758
Lissamphibia - Lepidosauria	-1.11372	-2.29049	0.063058	0.072546
Pan-Mammalia - Lepidosauria	-0.03038	-1.06692	1.006156	0.99999
Pan-Archosauria (excluding Crown Aves) - Lepidosauria	-0.71156	-1.21436	-0.20877	0.00149
Pan-Mammalia - Lissamphibia	1.083333	-0.39068	2.557344	0.2527
Pan-Archosauria (excluding Crown Aves) - Lissamphibia	0.402157	-0.76021	1.564523	0.870644
Pan-Archosauria (excluding Crown Aves) - Pan-Mammalia	-0.68118	-1.70133	0.338975	0.346708
**Intracolumnar heterogeneity by clade**	**diff**	**lwr**	**upr**	**p adj**
Lepidosauria - Crown Aves	-0.99577	-1.26124	-0.73029	0
Lissamphibia - Crown Aves	-1.0441	-1.62775	-0.46046	3.02E-05
Pan-Mammalia - Crown Aves	-0.26327	-0.77736	0.250821	0.612533
Pan-Archosauria (excluding Crown Aves) - Crown Aves	-0.12048	-0.36985	0.128895	0.663675
Lissamphibia - Lepidosauria	-0.04833	-0.63198	0.535309	0.999361
Pan-Mammalia - Lepidosauria	0.7325	0.21841	1.24659	0.001358
Pan-Archosauria (excluding Crown Aves) - Lepidosauria	0.875294	0.625924	1.124664	0
Pan-Mammalia - Lissamphibia	0.780833	0.049772	1.511894	0.03025
Pan-Archosauria (excluding Crown Aves) - Lissamphibia	0.923627	0.347132	1.500123	0.000228
Pan-Archosauria (excluding Crown Aves) - Pan-Mammalia	0.142794	-0.36317	0.648756	0.934029
**Region scores by Ecology**	**diff**	**lwr**	**upr**	**p adj**
AR-A	0.5495	-0.69677	1.795773	0.735611
SA-A	0.133452	-1.01578	1.28269	0.997599
T-A	-0.01432	-1.11445	1.085811	1
V-A	-0.12985	-1.30052	1.040817	0.997992
SA-AR	-0.41605	-1.22542	0.393329	0.609665
T-AR	-0.56382	-1.30181	0.174171	0.217984
V-AR	-0.67935	-1.51888	0.160178	0.170294
T-SA	-0.14777	-0.7065	0.410957	0.947465
V-SA	-0.26331	-0.95059	0.42398	0.823234
V-T	-0.11553	-0.71711	0.486045	0.983526
**Intracolumnar heterogeneity by ecology**	**diff**	**lwr**	**upr**	**p**
AR-A	-0.0295	-0.8102	0.7512	0.999972
SA-A	0.624405	-0.09551	1.344319	0.120874
T-A	0.515682	-0.17347	1.204833	0.23652
V-A	0.953677	0.220336	1.687017	0.004322
SA-AR	0.653905	0.146889	1.16092	0.004762
T-AR	0.545182	0.082885	1.007479	0.012419
V-AR	0.983177	0.457271	1.509082	1.18E-05
T-SA	-0.10872	-0.45873	0.241279	0.909045
V-SA	0.329272	-0.10126	0.759806	0.217061
V-T	0.437995	0.061148	0.814841	0.014304

Supplementary Table 9. Ancestral state estimations for region number at key nodes, modelled under different phylogenetic hypotheses. Letters correspond to Supplementary Figure 11. **A.** *Champsosaurus laramiensis* as a stem archosaur, *Dolichorhynchops osborni* as a stem reptile. **B.** *Champsosaurus laramiensis* as a stem reptile, *Dolichorhynchops osborni* as a stem archosaur. **C.** *Champsosaurus laramiensis* as a stem archosaur, *Dolichorhynchops osborni* as a stem archosaur. **D.** Both taxa removed

Phylogenetic hypothesis	Original (Fig. 3, Supplementary Figure 1)			A			B			C			D		
**State at node**	**2**	**3**	**4**	**2**	**3**	**4**	**2**	**3**	**4**	**2**	**3**	**4**	**2**	**3**	**4**
**Whole sample**	0.58	0.42	0	0.28	0.71	0.01	0.47	0.53	0	0.35	0.65	0	0.37	0.63	0
**Lissamphibia**	0.27	0.71	0.02	0.45	0.53	0.02	0.45	0.55	0	0.41	0.55	0.04	0.37	0.6	0.03
**Aminota**	0.3	0.7	0	0.19	0.81	0	0.23	0.77	0	0.17	0.83	0	0.17	0.83	0
**Synapsida**	0.19	0.81	0	0.22	0.78	0	0.12	0.87	0.01	0.13	0.87	0	0.1	0.87	0.03
**Eureptilia**	0.3	0.7	0	0.34	0.66	0	0.27	0.73	0	0.32	0.68	0	0.28	0.72	0
**Lepidosauria**	0.24	0.76	0	0.31	0.69	0	0.3	0.7	0	0.33	0.66	0.01	0.24	0.75	0.01
**Archosauromorpha**	0.15	0.85	0	0.32	0.68	0	0.18	0.82	0	0.31	0.69	0	0.09	0.91	0
**Pseudosuchia**	0.24	0.76	0	0.12	0.88	0	0.11	0.89	0	0.09	0.91	0	0.07	0.93	0
**Ornithodira**	0.29	0.71	0	0.18	0.82	0	0.18	0.82	0	0.12	0.88	0	0.13	0.87	0
**Dinosauria**	0.3	0.7	0	0.17	0.83	0	0.16	0.84	0	0.15	0.85	0	0.13	0.87	0
**Theropoda**	0.87	0.13	0	0.9	0.1	0	0.89	0.11	0	0.85	0.15	0	0.88	0.12	0
**Aves**	0.85	0.15	0	0.95	0.05	0	0.92	0.08	0	0.97	0.03	0	0.97	0.03	0
