# Supplementary material for: Evolution of the reptile spine reveals independent trajectories to axial skeletal complexity in amniotes
Source: Nat Commun. 2026 Apr 16;17:5304. doi: 10.1038/s41467-026-72071-x (PMC13269487; doi:10.1038/s41467-026-72071-x)
Supplement: Supplementary file 2 — Description of Additional Supplementary Files [file 41467_2026_72071_MOESM2_ESM.pdf]

Description of Additional Supplementary Files

Supplementary Data 1. Information on each specimen used in regionalization analyses.

Institutional abbreviations are as follows: UMZC = University Museum of Zoology, University of Cambridge; FMNH = Field Museum of Natural History, Chicago; USNM = Smithsonian National Museum of Natural History, Washington D.C.; TMM = Texas Memorial Museum, The University of Texas at Austin; CrocBase = https://osf.io/6zamj/;TCWC = The Biodiversity Research and Teaching Collections (Texas A&M University Biodiversity Research and Teaching Collections); YPM = The Peabody Museum of Natural History, Yale; UCMP = The University of California Museum of Paleontology, Berkeley; AMNH = American Museum of Natural History, New York; UMMP = The University of Michigan Museum of Paleontology**; SAM = Iziko South African Museum; NHMUK = The Natural History Museum, London; MCZ = Harvard Museum of Comparative Zoology; DMNS = Denver Museum of Nature & Science; CM = Carnegie Museum of Natural History, Pittsburgh; NMHU = Natural History Museum of Utah, Salt Lake City; UF = Florida Museum of Natural History; MVZ = Museum of Vertebrate Zoology, University of California, Berkeley; UMMZ = University of Michigan Museum of Zoology; CAS = California Academy of Sciences, San Francisco; PIMUZ = Palaeontological Museum of the University of Zurich; UA, Université d'Antananarivo, Antananarivo, Madagascar. * indicates specimen was downloaded from morphosource.org. Standard Morphosource agreement applies to all scans obtained from Morphosource. ** indicates specimen was downloaded from UMORF | University of Michigan Online Repository of Fossils https://umorf.ummp.lsa.umich.edu/wp/terms/.

Ecological category abbreviations are as follows: T = Terrestrial, V = Volant, AR = Arboreal, SA = Semi-aquatic, A = Fully aquatic

Supplementary Data 2. Sources for body mass data
