# Supplementary material for: Evolution of the reptile spine reveals independent trajectories to axial skeletal complexity in amniotes
Source: Nat Commun. 2026 Apr 16;17:5304. doi: 10.1038/s41467-026-72071-x (PMC13269487; doi:10.1038/s41467-026-72071-x)
Supplement: Supplementary file 5 — Reporting Summary [file 41467_2026_72071_MOESM5_ESM.pdf]

nature portfolio

Corresponding author(s): Lucy Roberts

Last updated by author(s): 2026/02/27

Reporting Summary

Nature Portfolio wishes to improve the reproducibility of the work that we publish. This form provides structure for consistency and transparency in reporting. For further information on Nature Portfolio policies, see our Editorial Policies and the Editorial Policy Checklist.

Statistics

For all statistical analyses, confirm that the following items are present in the figure legend, table legend, main text, or Methods section.

n/a	Confirmed	
☐	☒	The exact sample size (*n*) for each experimental group/condition, given as a discrete number and unit of measurement
☐	☒	A statement on whether measurements were taken from distinct samples or whether the same sample was measured repeatedly
☐	☒	The statistical test(s) used AND whether they are one- or two-sided<br>*Only common tests should be described solely by name; describe more complex techniques in the Methods section.*
☐	☒	A description of all covariates tested
☐	☒	A description of any assumptions or corrections, such as tests of normality and adjustment for multiple comparisons
☐	☒	A full description of the statistical parameters including central tendency (e.g. means) or other basic estimates (e.g. regression coefficient) AND variation (e.g. standard deviation) or associated estimates of uncertainty (e.g. confidence intervals)
☐	☒	For null hypothesis testing, the test statistic (e.g. *F*, *t*, *r*) with confidence intervals, effect sizes, degrees of freedom and *P* value noted<br>*Give P values as exact values whenever suitable.*
☒	☐	For Bayesian analysis, information on the choice of priors and Markov chain Monte Carlo settings
☒	☐	For hierarchical and complex designs, identification of the appropriate level for tests and full reporting of outcomes
☒	☐	Estimates of effect sizes (e.g. Cohen's *d*, Pearson's *r*), indicating how they were calculated

Our web collection on statistics for biologists contains articles on many of the points above.

Software and code

Policy information about availability of computer code

Data collection	Artec Studio 13 Professional was used to produce and process surface scans. Avizo (ver. 9) was used to process CT scan data.
Data analysis	R (4.2.0) in RStudio (2023.06.1) was used for all data analysis, using the packages geomorph (3.0.6), shapes (1.2.5), Morpho (2.11), regions (1.2), paleotree (3.3.25), phytools (1.6-16), ape (5.7-1), mvMORPH (1.1.7). All software and packages are publicly available. Custom code created to produce the results presented herein is available in github repository https://github.com/LucyEmmaRoberts/VertebralEvolution

For manuscripts utilizing custom algorithms or software that are central to the research but not yet described in published literature, software must be made available to editors and reviewers. We strongly encourage code deposition in a community repository (e.g. GitHub). See the Nature Portfolio guidelines for submitting code & software for further information.

Data

Policy information about availability of data

All manuscripts must include a data availability statement. This statement should provide the following information, where applicable:

- Accession codes, unique identifiers, or web links for publicly available datasets
- A description of any restrictions on data availability
- For clinical datasets or third party data, please ensure that the statement adheres to our policy

The landmark data generated in this study have been deposited in the github repository https://github.com/LucyEmmaRoberts/VertebralEvolution

Meshes and CT data generated for this study have been deposited on Morphosource under accession code 000601963
https://www.morphosource.org/projects/000601963?locale=en

Source data used to generate figure 1 and 2 are provided as a Source Data File. Data used to generate figures 3, 4 and 6 can be found in Supplementary Data 1. Data used to generate figure 5 is can be found in Supplementary Table 6.

Research involving human participants, their data, or biological material

Policy information about studies with human participants or human data. See also policy information about sex, gender (identity/presentation), and sexual orientation and race, ethnicity and racism.

Reporting on sex and gender	N/A no human participation or data used in this study.
Reporting on race, ethnicity, or other socially relevant groupings	N/A no human participation or data used in this study
Population characteristics	N/A no human participation or data used in this study
Recruitment	N/A no human participation or data used in this study
Ethics oversight	N/A no human participation or data used in this study

Note that full information on the approval of the study protocol must also be provided in the manuscript.

Field-specific reporting

Please select the one below that is the best fit for your research. If you are not sure, read the appropriate sections before making your selection.

☒ Life sciences ☐ Behavioural & social sciences ☐ Ecological, evolutionary & environmental sciences

For a reference copy of the document with all sections, see nature.com/documents/nr-reporting-summary-flat.pdf

Life sciences study design

All studies must disclose on these points even when the disclosure is negative.

Sample size	96 taxa were included in the analysis. Taxonomic sampling was determined by specimen availability and completeness. This sample includes individuals from each major clade under consideration. For each taxon every post-atlas-axis presacral vertebra was included in the analysis - the sample size of elements for each taxon was determined by the anatomy of each taxon. For each sampled taxon the number of sampled vertebrae varies between 17 and 42, resulting in a final overall sample of 2132 vertebral elements.
Data exclusions	The original taxonomic sample was larger (102 taxa). Following extensive sensitivity testing six individuals were removed due to completeness of the available sample of vertebral elements. In these sensitivity tests we took complete specimens and reiterated the analysis sequentially removing vertebral segments to gauge the tolerance of the methodology to data incompleteness.
Replication	We ran replicates using different numbers of landmarks, with different amounts of simulated data loss, and with different numbers of principal components in the regions analysis. Landmarks were chosen to be applies to homologous, unambiguous positions that can be fully replicated. All attempts at replication of the methodology were successful. We ran replicate analyses on 12 specimens of Sceloporus undulatus, two specimens of Crocodylus niloticus, Tomistoma schlegelii, Crocodylus moreletii and Osteolaemus tetraspis. We ran 28 replicates (7 replicates on 4 specimens) testing landmark number and choice. We ran 28 replicates (7 replicates on 4 specimens) to assess the impact of principal component reduction.
Randomization	N/A sampling of each vertebral column was determined by the individual biology of the specimen (regions analysis is within-column).
Blinding	N/A sampling of each vertebral column was determined by the individual biology of the specimen (regions analysis is within-column).

Reporting for specific materials, systems and methods

We require information from authors about some types of materials, experimental systems and methods used in many studies. Here, indicate whether each material, system or method listed is relevant to your study. If you are not sure if a list item applies to your research, read the appropriate section before selecting a response.

Materials & experimental systems

n/a	Involved in the study	
☒	☐	Antibodies
☒	☐	Eukaryotic cell lines
☐	☒	Palaeontology and archaeology
☒	☐	Animals and other organisms
☒	☐	Clinical data
☒	☐	Dual use research of concern
☒	☐	Plants

Methods

n/a	Involved in the study	
☒	☐	ChIP-seq
☒	☐	Flow cytometry
☒	☐	MRI-based neuroimaging

Palaeontology and Archaeology

Specimen provenance: We did not collect specimens for this study, all paleontological specimens are housed in museum collections. No permits were required, no specimens were moved, imported or exported. Specimen numbers and locations are available in Supplementary Data 1. All providence information is available at each institution.

Specimen deposition: N/A no specimens were collected for this study.

Dating methods: N/A no specimens were collected for this study.

☐ Tick this box to confirm that the raw and calibrated dates are available in the paper or in Supplementary Information.

Ethics oversight: No ethical approval was required. No new specimens were collected for this study. All specimen work was non-destructive (CT or surface scanning).

Note that full information on the approval of the study protocol must also be provided in the manuscript.

Plants

Seed stocks: N/A no plants were included in this study.

Novel plant genotypes: N/A no plants were included in this study.

Authentication: N/A no plants were included in this study.
