# Supplementary material for: Evolution of the reptile spine reveals independent trajectories to axial skeletal complexity in amniotes
Source: Nat Commun. 2026 Apr 16;17:5304. doi: 10.1038/s41467-026-72071-x (PMC13269487; doi:10.1038/s41467-026-72071-x)
Supplement: Supplementary file 6 — Transparent Peer Review file [file 41467_2026_72071_MOESM6_ESM.pdf]

nature portfolio

Peer Review File

Evolution of the reptile spine reveals independent trajectories to axial skeletal complexity in amniotes

Corresponding Author: Dr Lucy Roberts

This manuscript has been previously reviewed at another journal. This document only contains information relating to versions considered at Nature Communications.

This file contains all reviewer reports in order by version, followed by all author rebuttals in order by version.

Version 0:

Reviewer comments:

Reviewer #3

(Remarks to the Author)
I reviewed this manuscript previously at another journal and returned minor comments. The authors have address those and the concerns of other reviewers sufficiently as far as I am concerned.

(Remarks on code availability)

Reviewer #4

(Remarks to the Author)
This revised ms addresses all the earlier concerns by previous reviewers and presents a cleaner ms concerning axial regionalization in amniotes. I have only a few specific comments, listed by line number.
l90. Your statement that you sampled the majority of extant reptile clades is poorly defined here. Sampling a single bird samples the entire clade of Reptilia. I assume you mean a less inclusive taxonomic level. Would be clearer if you stated what this level is.
l90. The exclusion of snakes is curious given the authorship. Were they excluded for a particular reason, apart from a statement that they have been examined before? Did their inclusion alter the evolutionary patterns within Squamata? Some mammals were included even though they have also been studied before. Excluding snakes from this analysis forces the reader to assume their inclusion changes your results to the point this ms has a different conclusion. A very clear reason for excluding snakes needs to be made here.
l104. The sensitivity analyses cover a lot of variables. However, perhaps the largest variable of all, phylogeny, is not tested! What happens if rearrangements of stem reptiles is altered? The citation of Neenan et al. 2013 for the stem reptile topology is curious because they place Choristodera in a generally accepted position within Archosauromorpha yet this ms places them as a step reptile. The placement of Dolichorhyncops has a similar issue with Neenan et al. 2013 positioning ichthyosaurs and sauropterygians sister to the archosaur-lepidosaur clade, and many others placing sauropterygians as stem archosauromorphs (e.g. Schoch, Rainer R.; Sues, Hans-Dieter (24 June 2015). "A Middle Triassic stem-turtle and the evolution of the turtle body plan". Nature. 523 (7562): 584–587 and Simões, T. R.; Kammerer, C. F.; Caldwell, M. W.; Pierce, S. E. (2022). "Successive climate crises in the deep past drove the early evolution and radiation of reptiles". Science Advances. 8 (33) eabq1898.)
l154. How can you model selection vs drift vs pleiotropic effects in this system? You can test for support for particular models of phylogenetic change, but certainly not selection.
l191. To not suggest to cite my work, but other examples of cryptic axial regionalization have been recovered, like: Bui, H.-N. N., and H. C. E. Larsson. 2021. Development and evolution of regionalization within the avian axial column. Zoological Journal of the Linnean Society 191:302–321.
l217. Can you plot body size and these regionalization values. Without it, this statement will remain a qualitative possibility. For example, tt appears that the largest reptile, a sauropod, has only three regions and the largest theropods have only two regions. This seems to contradict this general statement that regionalization increases with body size.
l350. How were deformed vertebrae treated? Opening a few fossil vertebral scans shows a large degree of shearing, with

distorted bilateral and dorsoventral symmetries. Since this is a geometric morphometric analysis, how were these distortions treated? There is no mention of any retrodeformation of the fossil scan data. Without such a method, are these fossils even a valid addition to the analysis?
l614. Italicize Caelestiventus hanseni.
Extended Data Figure S9. I'm surprised that the boundary between dorsals 3-4 and dorsal 9-10 of Anzu does not reveal a regional boundary. The opposing centra have very different articular surface dimensions to the point they seem to not have been able to articulate in life! How were these obvious mismatches handled?
Please reveal my name to the authors should they have any questions concerning my review. Hans Larsson.

(Remarks on code availability)
The data is intact. But, see my comment about fossil retrodeformation above.

Version 1:

Reviewer comments:

Reviewer #4

(Remarks to the Author)
Most issues are well responded in this revision. I’m surprised the radical phylogenetic alternatives had little effect on node reconstructions, but perhaps this speaks to a greater generality of either sauropsids, the method, sensitivity of the method, or some combination of these.

My comment on the Extended Data Figure S9 concerning Anzu, as an example, seems to have been misinterpreted. Perhaps I was being a bit too sarcastic. The lack of articulation in the opposing centra of D3-4 and D9-10 is either a taphonomic artifact or misidentified vertebrae. Their lack of articulation should, with the geometric method, reveal a large enough difference between these pairs to identify a regional boundary. My comment here is only that in this case of taphonomic distortions not ameliorated by left-right symmetry retrodeformation, the geometric results still don’t identify a regional boundary where functionally (as in the vertebrae cannot fit together) there would be one. I think a clearer discussion on which specimens had to be retrodeformed would make this study more reproducible, perhaps as a supplementary table. For example, for Anzu, it was included in the analysis, so passed the incomplete specimen threshold, but how much editing was required for each vertebrae prior to measurement?

(Remarks on code availability)
All fine. Was nice to see the original dataset (scans) included in the supplementary data.

Open Access This Peer Review File is licensed under a Creative Commons Attribution 4.0 International License, which permits use, sharing, adaptation, distribution and reproduction in any medium or format, as long as you give appropriate credit to the original author(s) and the source, provide a link to the Creative Commons license, and indicate if changes were

made.
In cases where reviewers are anonymous, credit should be given to 'Anonymous Referee' and the source.
The images or other third party material in this Peer Review File are included in the article's Creative Commons license, unless indicated otherwise in a credit line to the material. If material is not included in the article's Creative Commons license and your intended use is not permitted by statutory regulation or exceeds the permitted use, you will need to obtain permission directly from the copyright holder.
To view a copy of this license, visit https://creativecommons.org/licenses/by/4.0/

Response to reviewers: Evolution of the reptile spine reveals independent trajectories to axial skeletal complexity in amniotes

Remarks to the Author in bold, responses from the Author in plain text

Reviewer #3 (Remarks to the Author):

I reviewed this manuscript previously at another journal and returned minor comments. The authors have address those and the concerns of other reviewers sufficiently as far as I am concerned.

We would like to thank reviewer #3 for their comments on this manuscript.

Reviewer #4 (Remarks to the Author):

This revised ms addresses all the earlier concerns by previous reviewers and presents a cleaner ms concerning axial regionalization in amniotes. I have only a few specific comments, listed by line number.

We would like to thank reviewer #4 for their comments on this manuscript.

l90. Your statement that you sampled the majority of extant reptile clades is poorly defined here. Sampling a single bird samples the entire clade of Reptilia. I assume you mean a less inclusive taxonomic level. Would be clearer if you stated what this level is.

We have added clarity to this portion of the manuscript, explicitly stating that we sampled extant crocodilians, birds and lepidosaurs (P3, L89-90).

l90. The exclusion of snakes is curious given the authorship. Were they excluded for a particular reason, apart from a statement that they have been examined before? Did their inclusion alter the evolutionary patterns within Squamata? Some mammals were included even though they have also been studied before. Excluding snakes from this analysis forces the reader to assume their inclusion changes your results to the point this ms has a different conclusion. A very clear reason for excluding snakes needs to be made here.

We excluded snakes because Head and Polly (2015) demonstrated that snakes and other snake-like squamates are just as regionalized as other reptiles (4 regions), we additionally discuss potential issues of high vertebral counts in analysis on page 9, lines 450-462. We have also added a clarifying statement in the main manuscript (P3, L92-94).

l104. The sensitivity analyses cover a lot of variables. However, perhaps the largest variable of all, phylogeny, is not tested! What happens if rearrangements of stem reptiles is altered? The citation of Neenan et al. 2013 for the stem reptile topology is curious because they place Choristodera in a generally accepted position within Archosauromorpha yet this ms places them as a step reptile. The placement of Dolichorhyncops has a similar issue with Neenan et al. 2013 positioning ichthyosaurs and sauropterygians sister to the archosaur-lepidosaur clade, and many others placing sauropterygians as stem archosauromorphs (e.g. Schoch, Rainer R.; Sues, Hans-Dieter (24 June 2015). "A Middle Triassic stem-turtle and the evolution of the turtle body plan". Nature. 523 (7562): 584–587 and Simões, T. R.; Kammerer, C. F.; Caldwell, M. W.; Pierce, S. E. (2022). "Successive climate crises in the deep past drove the early evolution and radiation of reptiles". Science Advances. 8 (33) eabq1898.)

We agree that the effect of the placement of taxa that have more controversial taxonomic positions should be tested. As such, we have added a new section to the sensitivity analysis (P29, L1216-1243), testing the effect on ancestral state estimations of four additional phylogenetic hypotheses:

A. Choristodera within Archosauromorpha; Sauropterigia as stem reptile
B. Choristodera as stem reptile; Sauropterigia within Archosauromorpha
C. Choristodera within Archosauromorpha; Sauropterigia within Archosauromorpha
D. Removing both *Champsosaurus laramiensis* and *Dolichorhynchops osborni*

We have also added a brief discussion of this in the main methods (P6, L304-306), and added the additional references as necessary (P14, L690-707; Extended Data Table S5). We have added a new extended data figure (S11) showing the results of ancestral state estimation using these alternative hypotheses, and a new extended data table (S11) showing the probabilities of ancestral states modelled at major nodes on each tree. We find little-to-no difference to our ancestral state estimations; the probability of a three-region state at the node uniting Archosauromorpha decreases if *C. laramiensis* is treated as an archosauromorph (to 68/69% from 82-91% under scenarios where *C. laramiensis* is treated as a stem reptile). Under every alterative phylogenetic hypothesis we maintain the same broad patterns of axial skeletal complexity across amniotes. The only node at which the modelled highest likelihood ancestral state changes under these alternative phylogenetic models is the root node uniting amniotes with our lissamphibian outgroup, which changes from a moderate probability of a two-region ancestral state (58% two-region, 42% three-region) to a moderate-high probability of a three-region ancestral state (28-47% two-region, 53-71% three-region). This has no impact on the results or conclusions we draw regarding axial skeletal evolution across amniotes and reptiles, only whether the evolution of a three-region axial skeleton from two regions is ancestral to the hypothetical common ancestor uniting Lissamphibia and Amniota.

l154. How can you model selection vs drift vs pleiotropic effects in this system? You can test for support for particular models of phylogenetic change, but certainly not selection.

We were using ‘selection’ in the context of OU models identifying shifting evolutionary optima/stabilising selection. However, we agree that it is not the appropriate term on page 4, lines 155 and 157, and have edited the text to reflect this.

l191. To not suggest to cite my work, but other examples of cryptic axial regionalization have been recovered, like: Bui, H.-N. N., and H. C. E. Larsson. 2021. Development and evolution of regionalization within the avian axial column. Zoological Journal of the Linnean Society 191:302–321.

We have added this citation (P4, L194; P13, L654-657). This publication was on the original list of relevant articles and was omitted by accident.

l217. Can you plot body size and these regionalization values. Without it, this statement will remain a qualitative possibility. For example, tt appears that the largest reptile, a sauropod, has only three regions and the largest theropods have only two regions. This seems to contradict this general statement that regionalization increases with body size.

This portion of the manuscript refers to heterogeneity, not regionalization. We state explicitly on page 5 line 216 that we do not recover a relationship between regionalization and body mass. We do find a significant positive relationship between body mass and heterogeneity (Fig 5C). We have added a plot showing logged body mass against regionalization in as an extended data figure (Extended Data Figure S12, P40), which we have added reference to in the main text (P4, L163; P4, L217).

l350. How were deformed vertebrae treated? Opening a few fossil vertebral scans shows a large degree of shearing, with distorted bilateral and dorsoventral symmetries. Since this is a geometric morphometric analysis, how were these distortions treated? There is no mention of any retrodeformation of the fossil scan data. Without such a method, are these fossils even a valid addition to the analysis?

Where appropriate we mirrored vertebral elements to ensure they were symmetrical (see ‘Damaged specimens’ in sensitivity analysis: P28-29, L1197-1212). Where this was not appropriate, or not possible due to large amounts of shearing or damage we omitted these vertebral elements. Where this resulted in too many missing elements (see ‘Incomplete specimens’ in sensitivity analysis: P27-28, L1104-1195) we omitted the whole specimen.

l614. Italicize *Caelestiventus hanseni*.

This correction has been made.

Extended Data Figure S9. I'm surprised that the boundary between dorsals 3-4 and dorsal 9-10 of Anzu does not reveal a regional boundary. The opposing centra have very different articular surface dimensions to the point they seem to not have been able to articulate in life! How were these obvious mismatches handled?

It is not uncommon for theropods to be less regionalized than expected – we see a similar phenomenon in *Ramphastos ambiguous* (Figure SB iv)*,* where the first two vertebrae in the third region are quite different in shape to the remaining elements in the region. However, in this analysis we quite deliberately made no *a-priori* assumptions regarding region boundaries. Regions reflect, by our definition, morphological gradients within the vertebral column, not necessarily groups of morphologically similar elements, though regions of vertebrae will generally be more morphologically similar to each other than to the elements of other regions. Region boundaries reflect shifts in morphological trajectories, not absolute similarity.

NCOMMS-25-45560A Response to reviewers comments

Reviewers comments are given in bold, author responses are given in plain text

Reviewer #4 (Remarks to the Author):

Most issues are well responded in this revision. I'm surprised the radical phylogenetic alternatives had little effect on node reconstructions, but perhaps this speaks to a greater generality of either sauropsids, the method, sensitivity of the method, or some combination of these.

We would like to thank the reviewer for the opportunity to demonstrate the robustness of the phylogenetic comparative methods used herein.

My comment on the Extended Data Figure S9 concerning Anzu, as an example, seems to have been misinterpreted. Perhaps I was being a bit too sarcastic. The lack of articulation in the opposing centra of D3-4 and D9-10 is either a taphonomic artifact or misidentified vertebrae. Their lack of articulation should, with the geometric method, reveal a large enough difference between these pairs to identify a regional boundary. My comment here is only that in this case of taphonomic distortions not ameliorated by left-right symmetry retrodeformation, the geometric results still don't identify a regional boundary where functionally (as in the vertebrae cannot fit together) there would be one. I think a clearer discussion on which specimens had to be retrodeformed would make this study more reproducible, perhaps as a supplementary table. For example, for Anzu, it was included in the analysis, so passed the incomplete specimen threshold, but how much editing was required for each vertebrae prior to measurement?

We have added a description of the specimens that we retrodeformed using the method described in the relevant section of the supplementary materials alongside a short description of the reason retrodeformation was required. We have clarified that the only retrodeformaion performed was the simple symmetrical method described in the supplementary methods e.g. to fix a missing transverse process, pre- or post-zygapophysis by mirroring the corresponding feature from the other side.

Reviewer #4 (Remarks on code availability):

All fine. Was nice to see the original dataset (scans) included in the supplementary data.
